# Immunoregulatory properties of cell free DNA

**DOI:** 10.1007/s00018-025-05862-y

**Published:** 2025-08-26

**Authors:** Francesca Ferrera, Tiziana Altosole, Samuele Tardito, Giuseppina Astone, Cinzia Bernardi, Alessia Parodi, Chiara Marini, Giuseppina Conteduca, Elena Cichero, Annalisa Salis, Leonardo Arpesella, Laura Camporesi, Andrea Lagazio, Valentina Rigo, Andrea Pigozzo, Gianluca Damonte, Paola Fossa, Daniela Fenoglio, Raffaele De Palma, Giorgio Inghirami, Gilberto Filaci

**Affiliations:** 1 Department of Internal Medicine (DIMI), University of Genoa, Genoa, Italy; 2 IRCCS, IRCCS San Martino, Genoa, Italy; 3 Pathology and Laboratory Medicine, Weill Cornell Medicine, New York, 10021 NY USA; 4 Biotherapy Unit, IRCCS San Martino, Genoa, Italy; 5 Department of Pharmacy, University of Genoa, Genoa, Italy; 6 Department of Experimental Medicine (DIMES), University of Genoa, Genoa, Italy; 7 Alfatest, S.r.l, Milan, Italy; 8 Center for Cancer & Immunology Research, Children’s National Hospital, Washington, 20010 DC USA

**Keywords:** Immune-modulations, Cell free DNAs, Autoimmune diseases, Tumors

## Abstract

Cell free DNA (cfDNA) is detectable at low concentrations in the plasma of healthy subjects and at high concentrations in disorders characterized by a high rate of necrotic events, such as tumors and vasculitis, leading to the release of necrotic DNA into the surrounding tissue and the bloodstream. Although cfDNA may act as a danger signal by binding to DNA sensors, triggering inflammation and immune responses, elevated cfDNA concentrations instead may exert immunoregulatory activities. Here, we show that exogenously administered cfDNA mediates immunoregulatory functions in vivo, in particular, it protects lupus-prone mice from disease progression and favors tumor growth in tumor-challenged mice. Our data suggest that cfDNA mediates immune regulatory activities by directly interacting with MHC class II molecules on antigen-presenting cells and through recruitment of regulatory T cells. This study unveils unprecedented biologic functions of cfDNA with significant pathogenic relevance and remarkable implications for the treatment of cancer patients.

**Supplementary Information:**

The online version contains supplementary material available at 10.1007/s00018-025-05862-y.

## Introduction

Cell free DNA (cfDNA) was first identified by Mandel and Metais in 1948 [1]. It is detectable in healthy plasma at concentrations ≤ 5 ng/ml [2], while higher concentrations are observed in patients affected by tumors or immune-based processes, like vasculitis [3].

Apoptotic and necrotic cells are the primary sources of cfDNA in the circulation [4], although there is also evidence for a mechanism of active secretion [5, 6]. Qualitative and quantitative alterations of cfDNA have been demonstrated in cancer patients suggesting that the analysis of cfDNA represents a useful biomarker in cancer [7, 8]. Conversely, the biologic role of cfDNA is controversial. Indeed, the discovery of the first DNA sensor, Toll-like receptor-9 (TLR-9), an endosomal pattern recognition receptor (PRR) specifically recognizing CpG rich DNA (typical of bacteria genome) [4], attributed to DNA immune-modulating functions. Thereafter, several DNA sensors have been identified in different cell types [5, 6]. Importantly, all of them are cytosolic and activate innate immunity and inflammatory reactions, leading to subsequent effector immune responses mainly by signaling through STING and NF-kB transducing factors [6]. These inflammatory DNA sensors are unlikely to represent the only receptors for cfDNA, since this would conflict with the need to tolerate autologous cfDNA in healthy conditions and pregnancy, a physiological condition in which cfDNA concentrations increase [9]. Moreover, the concept that cfDNA exerts only pro-inflammatory activities would not justify high cfDNA concentrations in conditions characterized by immune tolerance and variable degrees of immunodeficiency as tumors. To explain this controversy, we hypothesized that cfDNA can also exert immunoregulatory activities through interaction with still-unknown ligands: according with this scenario, recent evidences suggest that activation of the STING pathway can contribute to immune suppression by activating immunoregulatory mechanisms such as production of indoleamine 2,3 dioxygenase (IDO) [10, 11]. Here we address the issue concerning the immune-modulating effects of non-microbial cfDNA by investigating its immune regulatory activities identifying their potential molecular mediators and in vivo effects.

## Results and discussion

### cfDNA-mediated immune regulatory activities

Antigen-specific proliferation activity of carboxyfluorescein diacetate succinimidyl ester (CFDA-SE)-labeled splenocytes isolated from ovalbumin (OVA) hyper-immune mice was tested in vitro against different concentrations of OVA (1 ng/ml to 100 µg/ml) to obtain a dose-response curve. We repeated the same experiment pre-incubating splenocytes with Poly-C/poly-G (100 ng/ml) for 1 h at 37 °C. Antigen-specific proliferation was analyzed by CFDA-SE dilution assay in flow cytometry and live/dead T cells and APC evaluation was performed (Fig.S1). We found a significant inhibition of antigen specific proliferation in presence of Poly-C/poly-G (*p* = 0.02) and a positive correlation (*p* = 0.03) in proliferating cell percentage in presence or absence of Poly-C/poly-G suggesting for a possible competition for the binding site between antigen and Poly-C/poly-G. (Fig.S2).

**Fig. 1:**
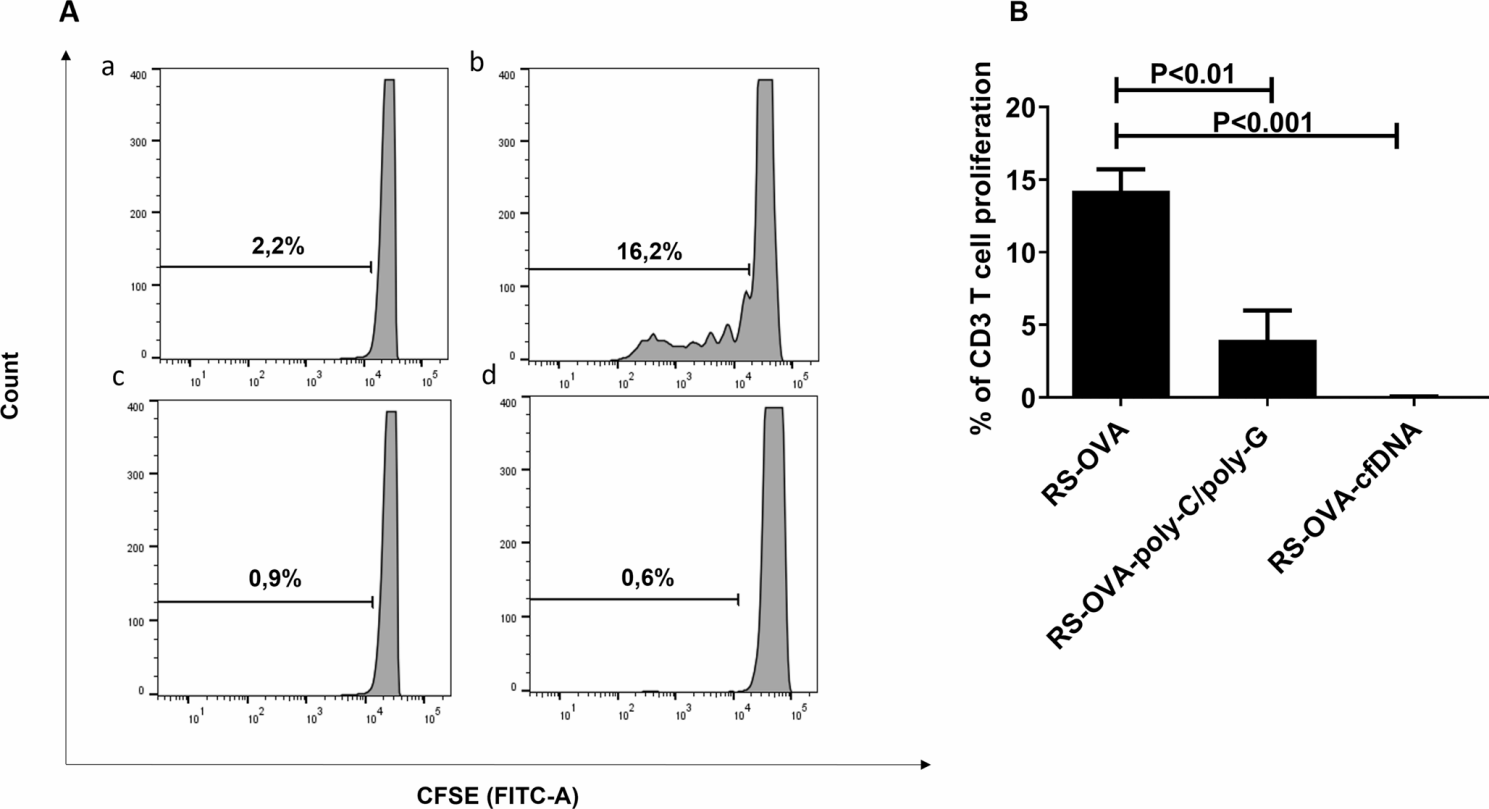
cfDNA inhibits antigen-specific T cell proliferation. (**A**) Representative OVA-specific proliferation assay performed with splenic T cells isolated from an OVA-immunized BALB/c mouse stimulated with: (a) irradiated autologous splenocytes; (b) irradiated autologous splenocytes plus OVA; (c) irradiated autologous splenocytes plus OVA and the poly-C/poly-G oligonucleotide (100 ng/ml); (d) irradiated autologous splenocytes plus OVA and cfDNA from cancer patients (2.5 ng/ml). (**B**) The graph shows the results of OVA-specific proliferation assays performed with splenic T cells from different OVA-immunized BALB/c mice incubated with irradiated autologous splenocytes plus OVA (left value); irradiated autologous splenocytes plus OVA and the poly-C/poly-G oligonucleotide (100 ng/ml) (middle value); irradiated autologous splenocytes plus OVA and cfDNA from cancer patients (2.5 ng/ml) (right value). Background values, corresponding to the percentage of proliferation of non-antigen stimulated splenic T cells from OVA-immunized BALB/c mice grown in the presence of irradiated autologous splenocytes without antigen, have been subtracted. Data are expressed as mean ± standard deviation. All values are expressed as mean ± standard error of the mean (SEM); three experiments, *N* = 3 mice per group; one-way ANOVA with additional Dunnett post-hoc test correction performed for groups multiple comparison.

Then we performed again the test with the highest OVA concentration (100 µg/ml) in presence or not of Poly-C/poly-G (100 ng/ml) or cfDNA purified from cancer patient’s sera (2.5 ng/ml). Antigen-specific proliferation was analyzed by CFDA-SE dilution assay in flow cytometry. The results from three independent experiments demonstrated that poly-C/poly-G DNA and cfDNA significantly inhibited OVA-specific T cell proliferation (Fig. 1 A-B; and Table S1), demonstrating also that DNA-mediated immune regulatory effects are not dependent on its source (synthetic versus biologic) or dimension (short synthetic oligonucleotides versus variably long DNA fragments from the circulation).

Hence, these results show that cfDNA impairs antigen-specific T cell proliferation, playing an immune regulatory activity.

We also tested the effects of the oligonucleotides in a mixed lymphocyte reaction (MLR). In brief, splenocytes from BWF1 mice were stained with CFDA-SE and used as Responder, splenocytes from C57BL mice were stained with Cell trace, irradiated and used as Stimulator. Responder were incubated with Stimulator (1:1) in the presence or not of poly-C/poly-G or poly-A/poly-T. Fig.S3 shows that Poly-C/Poly-G inhibited proliferation of BWF1 splenocytes in the MLR environment.

To better elucidate the mechanism by which cfDNA induces suppression of proliferation we analyzed if cfDNA could act by blocking CD3/CD28-mediated T cell proliferation. To this aim a CD3/CD28-mediate T cell proliferation assay was set up. As shown in Fig. S4, DNA didn’t modify significantly T cell proliferation induced by anti-CD3/CD28 both in the presence or not of APC demonstrating that cfDNA-mediated inhibition of T cell proliferation doesn’t act on the CD3/CD28 pathway.

To better characterize immune-regulatory activity of cfDNA we then look at the cytokines production by a macrophage cell line stimulated with Lipopolysaccharide (LPS) and treated or not with different concentrations of Poly-C/poly-G.

**Fig. 2:**
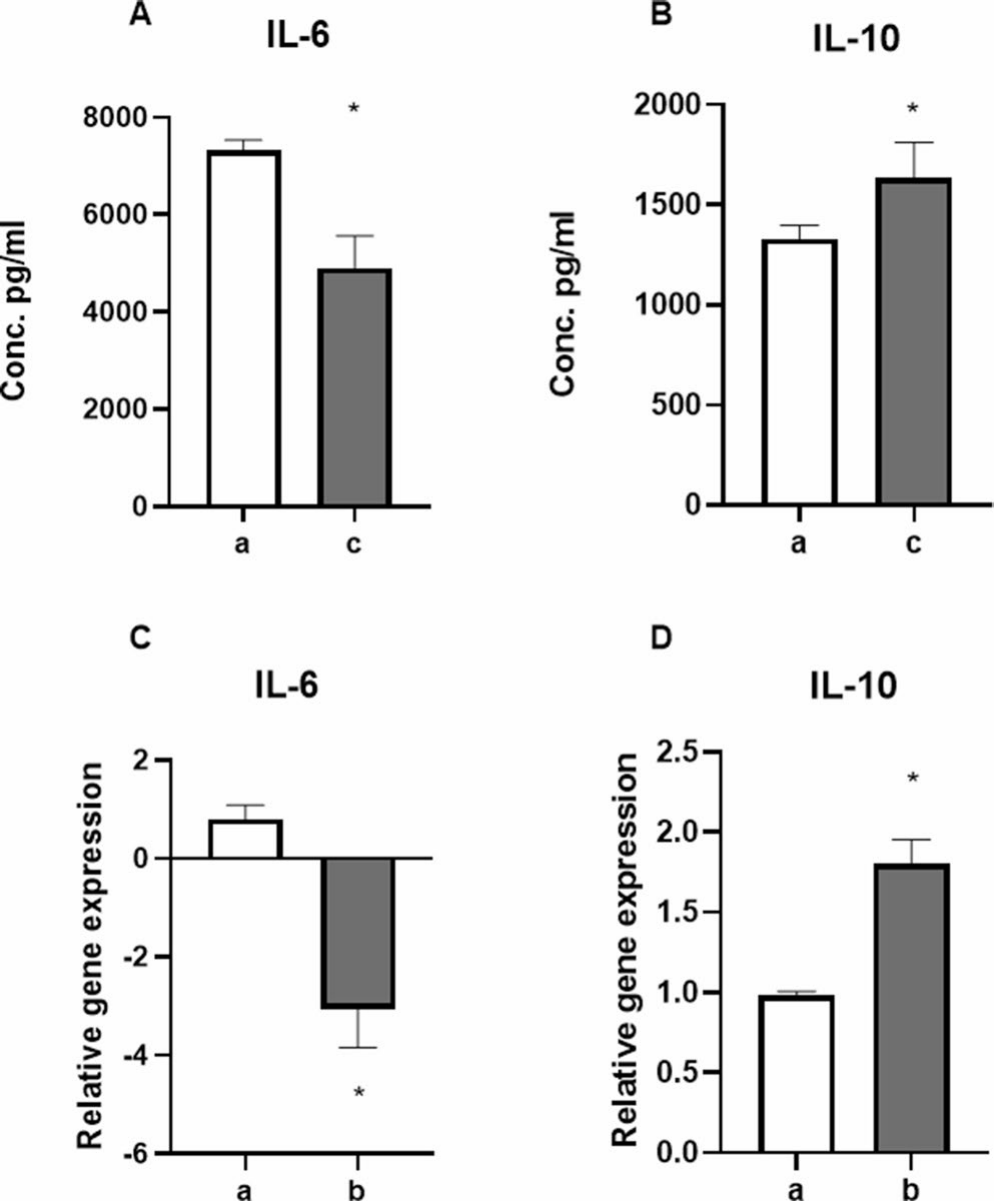
cfDNA modulates cytokines production in macrophages. Panels A and B: IL-6 (**A**) and IL-10 (**B**) cytokine levels in supernatants derived from RAW cells stimulated with LPS alone (a) or LPS and Poly-C/poly-G oligonucleotide (100 ng/ml) (b).Transcriptomic analysis of intracellular concentrations of mRNA coding for either IL6 (**C**) or IL10 (**D**) in LPS pre-activated PMJ2-PC macrophages treated (b) or not (a) with the poly-C/poly-G oligonucleotide (100 ng/ml), * *p*-value < 0.05

Production of IL-6, as representative pro-inflammatory cytokine, and IL-10, as representative tolerogenic cytokine, was evaluated analyzing protein concentrations in the supernatants by ELISA (Fig. 2 A-B) and intracellular copy number of specific coding mRNAs by Real time PCR (Fig. 2 C-D).

Data of both analyses showed a significative decrease of IL-6 production associated with a significative increase of IL-10 production in samples treated with the Poly-C/poly-G oligonucleotide (Fig. 2), confirming the immune regulatory activity of cfDNA.

### cfDNA binds to murine MHC class II (I-A) molecules

**Fig. 3:**
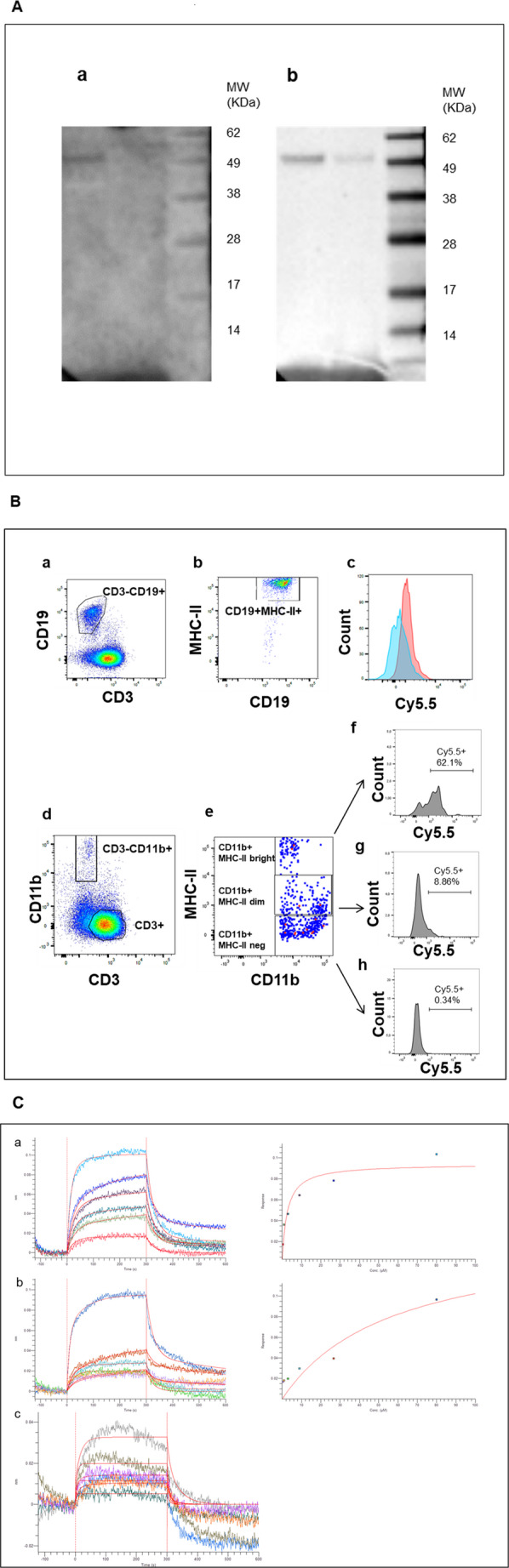
cfDNA binds to MHC class II molecules. (**A**) Lysates of splenocytes from a BWF1 mouse at different concentrations (150 ug lane 1 and 75 ug lane 2) were run on SDS-PAGE gel and then blotted. The membranes were incubated with either the poly-C/poly-G oligonucleotide followed by an anti-dsDNA mAb (a) or an anti-I-A^d^ MHC class II mAb (b). Bands with the identical molecular weight were detected in both conditions. The molecular standards are shown on the right side of each blot (Invitrogen Seeblue LC5925). (**B**) Binding of Cy5.5-labeled poly-C/poly-G oligonucleotide to MHC class II + B lymphocytes (a, b,c,) and monocytes (d, e,f, g,h) from IL-2 stimulated splenocytes of C57BL mice. (a) The gate showing B cells has been identified by splenocytes incubation with an anti-CD3 and an anti-CD19 mAbs; (b) Expression of MHC class II by B lymphocytes; (c) Staining of B cells incubated (red peak) or not (blue peak) with the Cy5.5-labeled poly-C/poly-G oligonucleotide; (d) The gate showing monocytes has been identified by splenocytes incubation with an anti-CD3 and an anti-CD11b mAbs; (e) Expression of MHC class II by monocytes: the upper, middle and lower squares show cells with high (CD11b + MHC-II bright), intermediate (CD11b + MHC-II dim) and absent (CD11b + MHC-II neg) MHC class II expression, respectively; (f) Staining of MHC Class II high monocytes with the Cy5.5-labeled poly-C/poly-G oligonucleotide; (g) Staining of MHC Class II intermediate monocytes with the Cy5.5-labeled poly-C/poly-G oligonucleotide; (h) Staining of MHC Class II negative monocytes with the Cy5.5-labeled poly-C/poly-G oligonucleotide. (**C**) BLI at different concentration of (a) I-A^d^ class II monomer, (b) I-A^b^ class II monomer and (c) H-2K^b^ class I monomer. It was possible to generate excellent fitting curves based on MHC monomer concentrations for MHC class II but not for MHC class I monomers

Since cfDNA inhibited the antigen-specific T cell proliferation, a process in which MHC molecules play a central role in mediating the antigen presentation, we then investigated possible molecular interactions between cfDNA and MHC class II molecules expressed by mouse APC. To this aim, lysates from splenocytes of BWF1 mice, expressing I-A^d^ I-E^u^ MHC class II antigens, were blotted on a nitrocellulose membrane after gel electrophoresis and then incubated with either an anti-I-A^d^ MHC class II monoclonal antibody (mAb) or the poly-C/poly-G synthetic oligonucleotide followed by an anti-dsDNA mAb. Bands with identical molecular weight were detected in both conditions (Fig. 3A), a finding suggesting the occurrence of a binding between MHC class II molecules and cfDNA.

To corroborate this observation, splenocytes from C57BL mice were incubated with a Cy5.5-labeled poly-C/poly-G oligonucleotide. Figure 3B shows that B lymphocytes (panels a-c) were stained by the Cy5.5-labeled poly-C/poly-G oligonucleotide, and that monocytes (panels d-f) with high MHC class II molecule expression had an increased cfDNA binding (in terms of mean fluorescence intensity) suggesting that MHC class II molecules could be ligands for cfDNA. To support these findings, a bio-layer interferometry (BLI) analysis was performed using I-A^d^ and I-A^b^ biotinylated monomers (kindly provided by NIH Tetramer Core Facility) and the poly-C/poly-G oligonucleotide ligand. A H-2K^b^ (MHC class I molecule) biotinylated monomer was used as a control. Robust interactions between the oligonucleotide and each of the MHC class II monomers were demonstrated by a dissociation constant (KD) in the micromolar range, while no specific binding was detected for the control MHC class I monomer. These findings assess the binding activity of MHC class II molecules for cfDNA (Fig. 3 C; and Table S2).

These assessment finds further support by the following observations. Figure 1 shows the inhibiting activity on T cell proliferation exerted by cfDNA when co-incubated with the specific antigen, suggesting a possible reciprocal competition when the antigen and the oligonucleotide are contemporarily exposed to their putative binding site (i.e. the groove of MHC II molecule). We repeated the T cell proliferation experiment preincubating antigen presenting cells with an excess of the antigen hypothesizing that in this condition the inhibitory effect mediated by the poly-C/poly-G could be hampered in the case of an identical binding site between the two different molecules. Indeed the results of Fig.S5 demonstrated absence of inhibitory activity by the poly-C/poly-G oligonucleotide in this experimental conditions.

Moreover a binding inhibition test was performed on RAW cells using a fluorescent Alexa fluor 488 (AF488)-labeled poly-C/poly-G oligonucleotide. The fluorescent oligonucleotide, at the different concentrations of (10, 5, 1 or 0,5 ug/ml) was added to cells in the presence or not of the anti-mouse-I-Ad MHC class II mAbM5/114.15.2 (2 µg/ml) or if its isotype control antibody. Cells were then analyzed by a Flow cytometer BD FACSCanto II flow cytometer (BD) using FACS DIVA (BD) software. Figure S6 shows that RAW cells incubated with both labeled poly-C/poly-G and anti-mouse -I-Ad MHC class II mAb displayed a statistically significant (*p* = 0.05) lower MFI respect to cells incubated with labeled poly-C/poly-G only or in combination with isotype control mAb. We also found that fluorescence intensity trend correlates with that of cells treated with labeled poly-C/poly-G only (*p* = 0.03) showing that the concentrations of the poly-C/poly-G oligonucleotide correlates with changes in the binding of the anti-mouse-I-Ad MHC class II mAb. Importantly, since the used anti-mouse-I-Ad MHC class II mAb is specific for the polymorphic determinants of I-Ad MHC class II molecules, these data strongly suggest that the competition for the binding site between the two agents occurs at the level of the binding site for the antigen [12].

At last, we performed a binding experiment on a MHC class II knockout macrophages cell line. PMJ-PC (MHC-II high) and PMJ-R (MHC-II low) macrophages cells were incubated with an AF488-labeled Poly-C/Poly-G oligonucleotide (100 ng/ml) and then analyzed by a Flow cytometer BD FACSCanto II flow cytometer (BD) using FACS DIVA (BD) software. Fig.S7 shows that PMJ-PC cells, having higher expression of MHC-II molecules, display a higher binding of the Poly-C/Poly-G oligonucleotide.

The above cited data suggest that cfDNA binds to MHC class II molecules. In order to further define the possible cfDNA binding site on MHC class II molecules, we performed docking analyses. We first built a three-dimensional model of the MHC class II protein by matching the coordinates of the corresponding alfa and beta chains, available at the Protein Data Bank [13] as pdb codes 1IEB and 1MUJ, respectively.

**Fig. 4:**
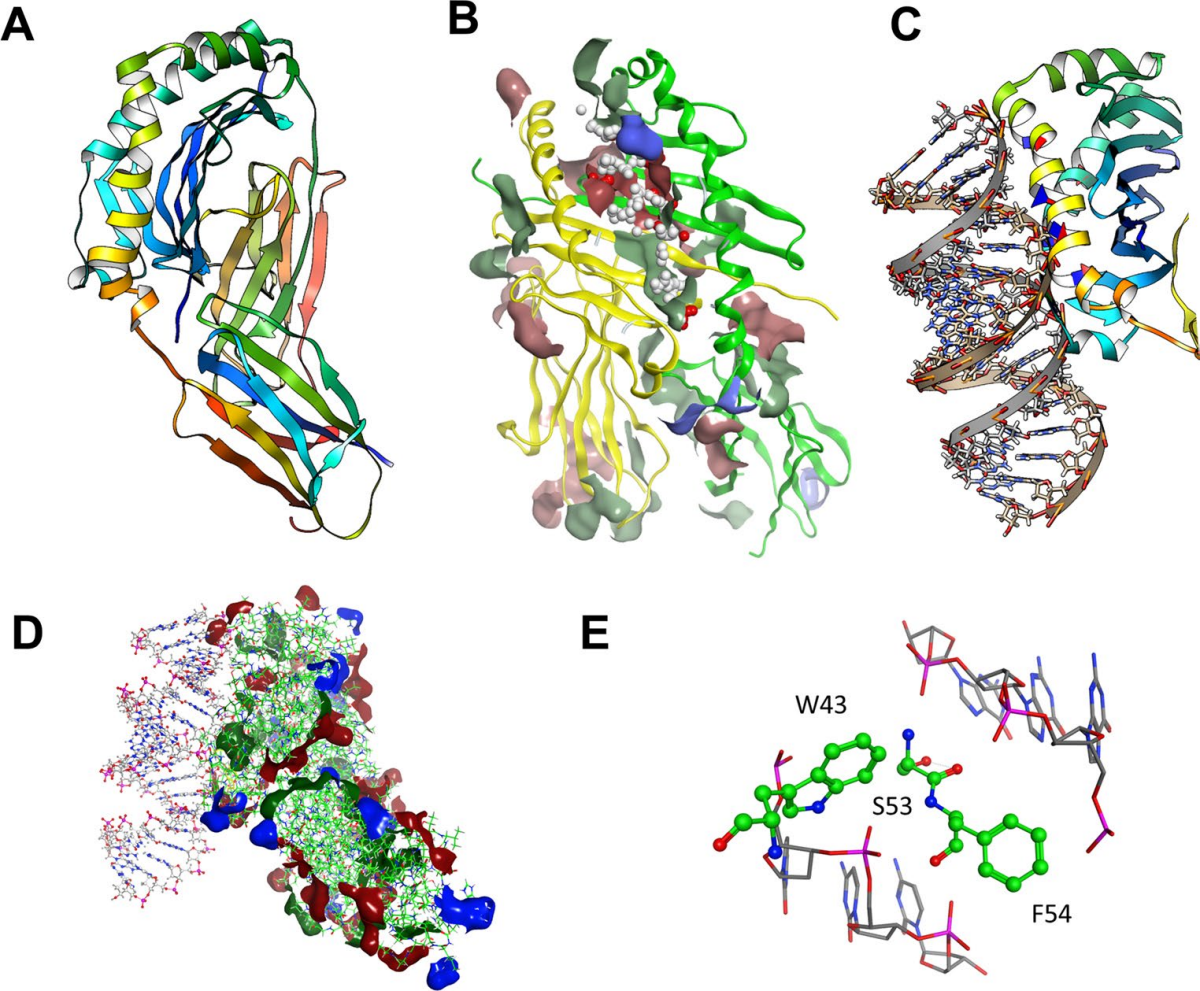
Docking analysis on the MHC class II poly-C/poly-G complex. (**A**) Three-dimensional model of the MHC class II complex herein investigated. Alpha helix and beta sheets are colored in red and yellow, respectively. (**B**) The best-scored binding site at the MHC class II complex is depicted by red and grey spheres, together with the patch areas calculated by the patch analyzer module implemented in MOE. (**C**) Docking of poly-C/poly-G at the MHC class II model surface. (**D**) Most relevant patch areas identified by molecular modeling analyses. (**E**) Key contacts related to the docking of poly-C/poly-G at the MHC class II model

The geometry of the obtained model refined by energy minimization methods led to the final model depicted in Fig. 4A. A preliminary evaluation of the most relevant cavities of the protein, which could be involved in ligand binding and/or in protein-protein contacts, was performed by running the Site Finder module implemented in the MOE software, (as we previously successfully did in other case studies) [14]. The purpose of Site Finder is to calculate possible active sites in a receptor from the 3D atomic coordinates of the receptor itself. Such a calculation is useful for guiding site-directed mutagenesis experiments as well as for exploring ligand-binding sites.

MOE’s Site Finder falls into the category of geometric methods in the search of putative binding site, since no energy models are used. Instead, the relative positions and accessibility of the receptor atoms are considered, along with a rough classification of chemical type. The Site Finder methodology revolves around the development and matching with the protein of convex hulls, namely Alpha Spheres, featuring a different polarity profile. In brief, the method acts as follows: (i) Identify regions of tight atomic packing, also on the surface of the protein. (ii) Filter out sites that are “too exposed” to solvent and retain all the others which are further classified based on their hydrophobic/hydrophilic properties. This is made by collecting a variable number of the aforementioned alpha spheres, which properly fit any putative site.

According to our results, the best-scored cavity was delimited by I7, Q9, E11, F22, F24, I31, F32, W43, A52, S53, F54, G58, A59, N62, V65, D66, N69 at the alpha chain and by F11, G13, Y26, T28, Y30, H47, W61, I67, R70, T71, E74, T77, V78, H81, N82, P85, E86, T89 within the beta chain. A schematic representation of the hydrophilic and hydrophobic areas of this crevice is shown respectively by red and grey spheres in Fig. 4B.

To refine and confirm these results, we explored our protein model’s surface to identify any region highly prone to protein aggregation or supporting protein-protein binding sites (namely patch areas). The final data revealed five patch areas, numbered as patch 1–5, exhibiting negatively-charged (red), or positively-charged (blue) or hydrophobic (green) surfaces, as shown in Fig. 4B. Specific properties of the aforementioned patches are summarized and listed in Table 1.

**Table 1:** Electrostatic profile of surface area of the most relevant patch locations obtained by molecular modeling analyses

Patch Number	Patch type	Area (Å^2^)	Residues	
			Alpha chain	Beta chain
1	Negatively-charged	140	Q9, E11, G58, A61, N62, V65, D66	F11, Y26, T28, T71, E74, V78
2	Hydrophobic	130	F22, F24, I31, F32, W43, A52, S53, F54	T77, V78, P85, T89
3	Hydrophobic	110	--	Y30, H47, Y60, W61, Q64, I67
4	Positively-charged	70	--	I67, R70
5	Positively-charged	60	E47, K50, F51	R92

Notably, these results enlightened some pockets of the modelled protein including the same regions detected by the aforementioned calculations with the site finder analysis (Fig. 4B). This approach supported the following molecular docking simulations with poly-C/poly-G to be performed at this region of the macromolecule.

In addition, the presence of the two positively-charged areas (4 and 5), which were the only couple of anchoring points for the negatively-charged phosphate groups of poly-C/poly-G detected at the MHC-II surface, defined the corresponding binding domain. Docking calculations revealed the putative binding mode of poly-C/poly-G at the MHC-II surface (Fig. 4 C), relying mainly on electrostatic contacts between the aforementioned phosphate groups of poly-C/poly-G and the positively charged 4 and 5 domains. Lastly, H-bond contacts were detected between poly-C/poly-G phosphate groups and the backbone of the alpha chain residues W43, S53 and F54 (patch 2) (Fig. 4D and E). Hence, model analysis strongly supports a putative binding between cfDNA and MHC class II molecules identifying the areas of molecular interaction in the antigenic groove.

### Effects of cfDNA-MHC class II interaction on gene expression

**Fig. 5:**
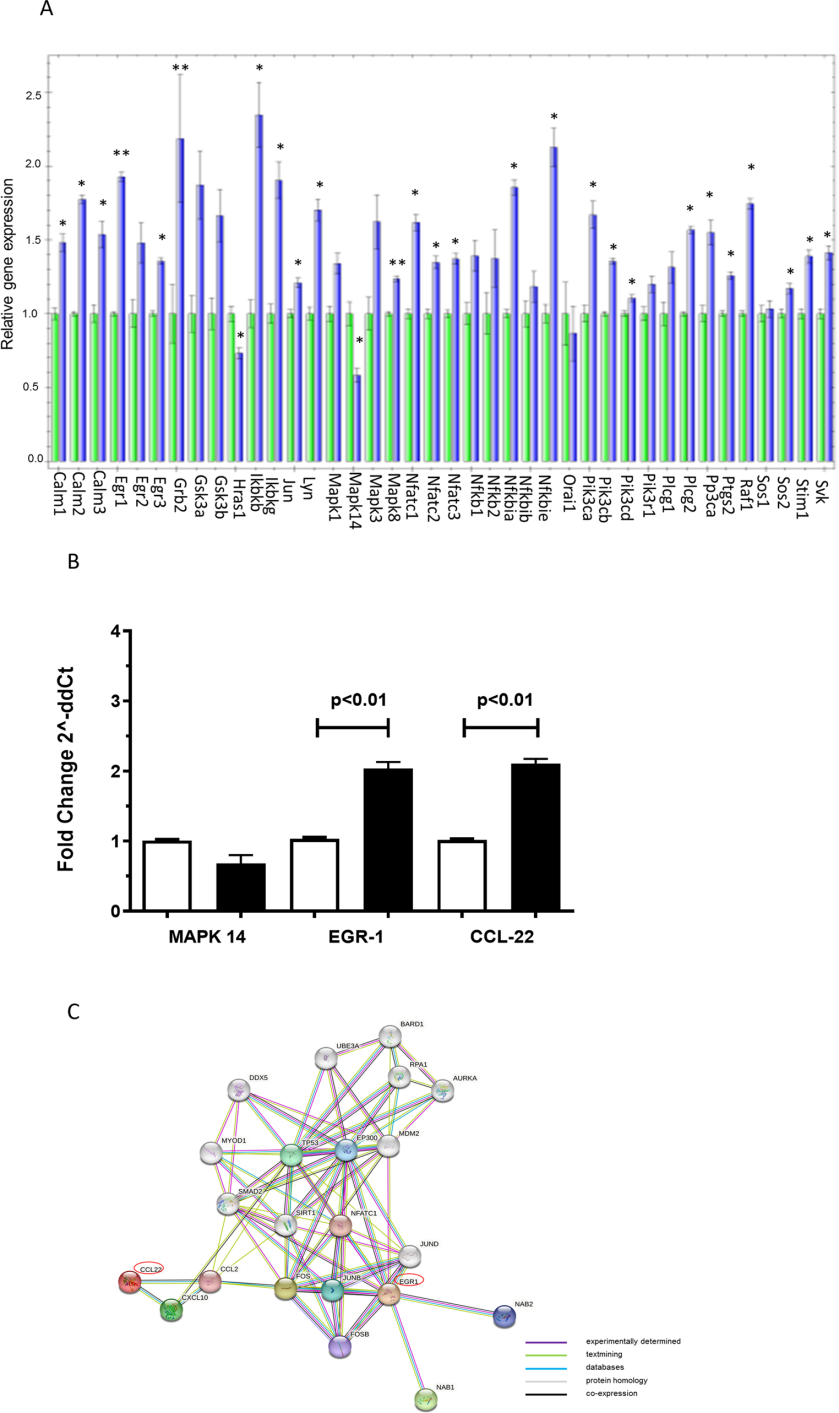
cfDNA-induced variation in gene expression. (**A**) 40 genes analyzed by Bio-Rad iQ5 analysis in LPS pre-activated PMJ2-PC macrophages treated (blue bar) or not (green bar) with the poly-C/poly-G oligonucleotide (1 µg/ml for 3 h), * *p*-value < 0.05 and ** *p*-value < 0.001; (**B**) Quantitative PCR analysis on mRNAs coding for *MAPK-14*,* EGR-1*, and *CCL22* in LPS pre-activated PMJ2-PC macrophages treated (black bar) or not (white bar) with the poly-C/poly-G oligonucleotide (1 µg/ml for 3 h). Data are expressed as fold change (2^− ΔΔCT^) normalized with GAPDH. Data are the mean of 3 concordant experiments; Student paired T test (**C**) Network analysis using the STRING-DB software of differentially expressed genes (*EGR-1* and *CCL22*) between pre-activated PMJ2-PC macrophages treated or not with the poly-C/poly-G oligonucleotide. *EGR-1* and *CCL22* genes are showed in red circles

The propensity of cfDNA to bind MHC class II molecules in the groove may explain cfDNA-mediated inhibition of antigen presentation based on possible binding competition with the antigenic peptide. However, we cannot exclude the possibility that cfDNA, binding to MHC class II molecules, may activate intracellular signaling pathways modifying the gene expression profile of APC, i.e., LAG3-MHC class II interactions [15, 16] as recently demonstrated [17]. Thus, we searched for transcriptional modulations induced by cfDNA on APC. LPS pre-activated PMJ2-PC cells (peritoneal macrophages derived from C57BL/6J mice) were incubated with poly-C/poly-G oligonucleotide and the expression of 40 genes was analyzed using Bio-Rad iQ5 pre-designed panel of pathway-specific genes. We observed that *Egr-1*, *Grb2*, *Ikbkb*, *Nfkbie* genes were up regulated (fold change ≥ 2.0) and *MAPK 14* was down regulated (fold change ≤ 0.5) by poly-C/poly-G oligonucleotide (Fig. 5 A; and Table S3). We then cross-validated these data by qPCR. To this end, we included the C-C motif ligand 22 (*CCL22*) gene since it is involved in the migration of T regulatory cells induced by macrophages. The qPCR results confirmed increased expression only of *Egr-1* and *CCL22* (fold change of ≥ 2.0) but not the downmodulation of *MAPK14* gene (Fig. 5B).

To assess the biological connection between the differentially expressed genes, we performed a network analysis using the STRING-DB software [18]. We extended the network to assess likely indirect interactions between differentially expressed genes. The resulting network, reported in Fig. 5 C, showed that *CCL22* and *Egr-1* may interact with a subset of other gene products, including *TP53*,* SIRT1*,* ESR1*,* EP300*,* FOS*,* MDM2* and *SMAD2*, taking part of a pattern of molecules involved in macrophages polarization from M1 to M2 subtype and in resistance of the tumor microenvironment to infiltration by tumor-specific lymphocytes [19–21].

### In vivo cfDNA homing and effects

We wonder whether the above-reported immune regulatory effects of cfDNA might be detected in vivo in conditions associated with elevated cfDNA concentrations. We reasoned that cfDNA-mediated immune regulation could be protective for inflammatory diseases but could be a cause of worsening for cancer. Based on this, we addressed the issue using two opposite experimental models, one representative for a chronic inflammatory condition (BWF1 lupus-prone mice, an experimental model of systemic lupus erythematosus, SLE), and the other one representative for cancer (B16F10 melanoma challenged C57BL mice). In our experiments we used cfDNA in the absence of any carrier molecule to avoid any interference related to the immune modulating effect mediated by the carrier and promotion of endocytosis. Preliminarily, we defined the amount of DNA to be injected to reproduce cfDNA levels comparable to those observed in SLE or cancer patients [3, 4, 7]. We found that intravenous injection of 10 µg of the poly-C/poly-G oligonucleotide induced a ≈ 5-fold increase of cfDNA concentrations (from 22.9 ng/ml at baseline to 187.31 ng/ml after 10 min from DNA administration, Table S4), producing cfDNA concentrations in mice similar to those detected in patients.

**Fig. 6:**
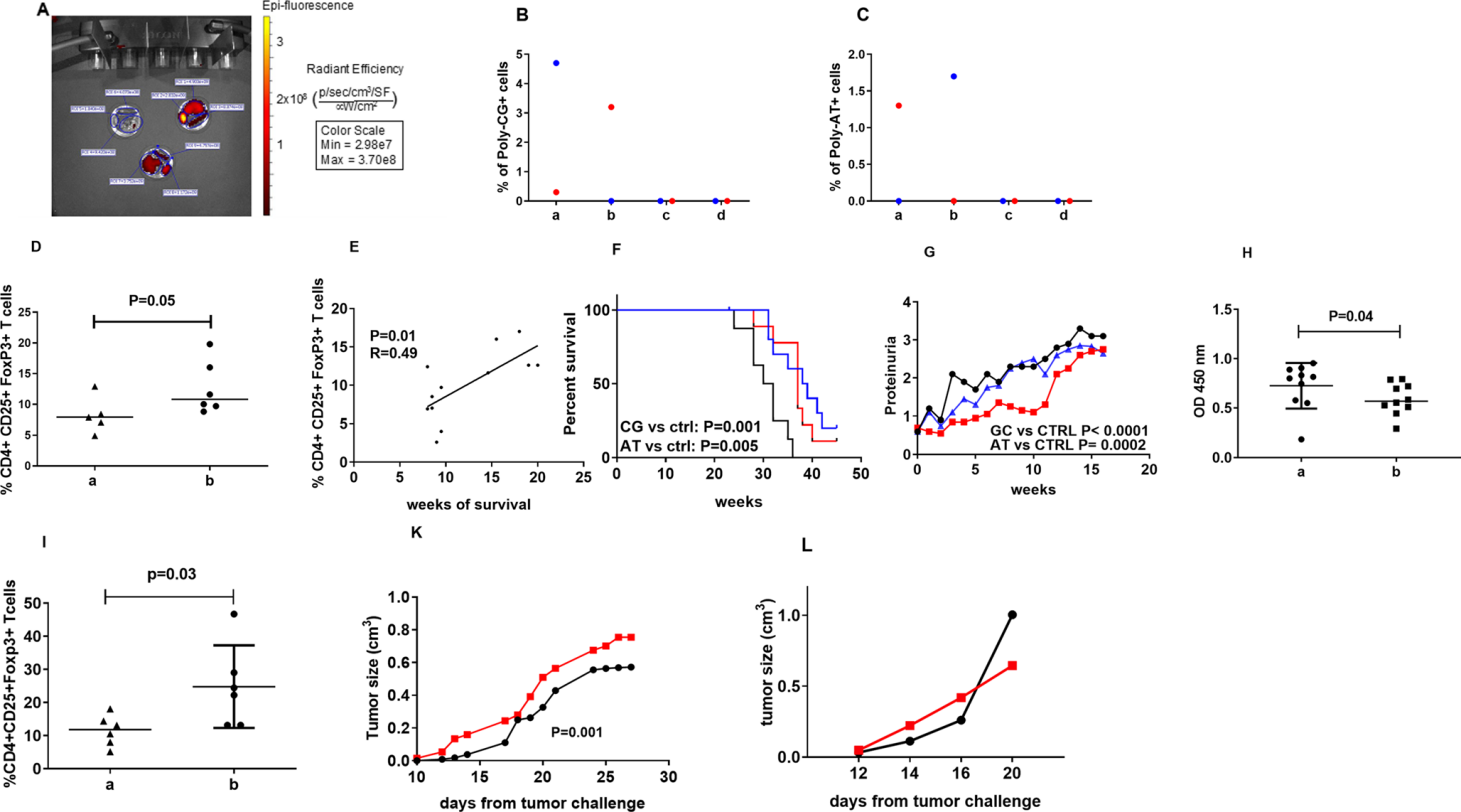
cfDNA effects in BWF1 SLE-prone (A-H) and in tumor challenged C57Bl (I-L) mice. (**A**) In Vivo Imaging System scanning, performed on spleens, kidneys and livers of BWF1 mice organs after 120 min from i.v. administration of the Cy5.5-labeled poly-C/poly-G oligonucleotide. (**B**) Percentage of Cy5.5-labeled poly-C/poly-G oligonucleotide labeled cells among (a) B lymphocytes, (b) macrophages, (c) T cells and (d) NK cells from splenocytes of Cy5.5-labeled poly-C/poly-G oligonucleotide administered mice (two mice, each color identifies data collected by each single mouse). (**C**) Percentage of Cy5.5-labeled poly-A/poly-T oligonucleotide labeled cells among (a) B lymphocytes, (b) macrophages, (c) T cells and (d) NK cells from splenocytes of Cy5.5-labeled poly-A/poly-T oligonucleotide administered mice (two mice, each color identifies data collected by each single mouse). (**D**) Frequency of CD4 + FoxP3 + CD25 + Treg among splenocytes from BWF1 SLE-prone mice untreated (a) or weekly i.v. administered with the poly-C/poly-G oligonucleotide (b). Student T test. (**E**) Correlation between survival and frequency of CD4 + FoxP3 + CD25 + Treg among splenocytes from BWF1 SLE-prone mice weekly i.v. administered with either the poly-C/poly-G or the poly-A/poly-T oligonucleotide. (**F**) Survival and (**G**) proteinuria levels of BWF1 SLE-prone mice untreated (black line) or weekly i.v. administered with either poly-C/poly-G (red line) or poly-A/poly-T (blue line) oligonucleotides (10 mice/group). The figure shows the mean of values achieved in one of two concordant experiments. Long-Rank (Mantel-Cox) Test for survival and 2way Anova Test for proteinuria. (**H**) Circulating anti-dsDNA antibody concentrations in untreated (a) and DNA administered (b) mice. Student T test. (**I**) Frequency of intratumoral CD4 + FoxP3 + CD25 + Treg from B16 melanoma induced C57BL mice untreated (a) or weekly i.v. administered with the poly-C/poly-G oligonucleotide (b) 6 mice/group, Student T test. (**K**) Tumor growth in B16F10 melanoma challenged C57BL mice administered (red line) or not (black line) with the poly-C/poly-G oligonucleotide. The figure shows the mean of values achieved in one of two concordant experiments. 7 mice/group, 2way Anova Test (**L**) Tumor growth in B16F10 melanoma challenged MHC class II KO C57BL mice administered (red line) or not (black line) with the poly-C/poly-G oligonucleotide. The figure shows the mean of values achieved in one experiment. 5 mice/group

To investigate in vivo cfDNA effects, we first focused on searching for the organ(s) where cfDNA mainly distributes. Ex vivo imaging was performed on organs extracted from BWF1 mice administered i.v. with Cy5.5-conjugated poly-C/poly-G oligonucleotides (10 µg). The analyses performed 120 min after DNA injection using the In Vivo Imaging System (IVIS) scanning (Perkin-Elmer, Walthman, MA) revealed that cfDNA localized mainly in the spleen, kidney and liver (Fig. 6 A).

Then, splenocytes from Cy5.5-conjugated poly-C/poly-G oligonucleotides i.v. injected mice were collected and analyzed by flow cytometry to unveil Cy5.5 fluorescence emission (indicative for Cy5.5-conjugated poly-C/poly-G binding), to get insights on the immune cells directly responsible for cfDNA uptake. The analysis showed that cfDNA was associated only to B lymphocytes and macrophages (Fig. 6B), which are cells expressing high levels of MHC class II molecules. To verify whether cfDNA protective effects were sequence-dependent, the experiment was replicated using 20 bp poly-A/poly-T oligonucleotide (constituted by a filament containing 20 adenines and a complementary filament containing 20 thymidines, 10 µg/injection), achieving consistent results (Fig. 6 C). These findings confirmed that in vivo cfDNA binds preferentially to MHC class II positive cells belonging to the pool of professional antigen presenting cells (APC).

Next, we focused on the Treg compartment, postulating that cfDNA-driven signals could contribute to their recruitment through CCL22 increased secretion by macrophages. BWF1 SLE-prone mice were weekly administered with DNA until they developed either nephritis with proteinuria level > 3 (corresponding to > 300 mg/dl) or ascites. The frequency of splenic CD4 + Treg was comparatively analyzed in DNA-treated mice and in controls. A significantly increased Treg frequency was observed in poly-C/poly-G treated mice with respect to control mice (*p* = 0.05) (Fig. 6D). Importantly, Treg frequency positively correlated with survival (*p* = 0.01) (Fig. 6E).

Finally, we investigated the relationship between increased cfDNA concentration and disease outcome, in line with the data generated by oligonucleotides conjugated to human gamma globulins and the reduction in vitro of anti-DNA antibody production mediated by an unknown mechanism [22]. DNA-treated mice showed significantly prolonged survival concerning to control mice (*p* = 0.001) in multiple experiments (Fig. 6 F), suggesting that the increased cfDNA concentrations could impact on the pathogenic mechanisms of disease likely through an immune regulatory effect. Accordingly, significantly lower proteinuria levels (Fig. 6G) and serum concentration of anti-dsDNA antibodies (Fig. 6H) were observed in DNA-treated mice than in controls, with a delayed development of nephritis (the major inflammatory manifestation in murine SLE) in the presence of high concentrations of cfDNA.

Collectively, these vivo data supports the notion that cfDNA exerts in vivo immune regulatory functions, leading to a protective effect in chronic inflammatory diseases, and that this effect is not linked to any specific nucleotide sequence. We reasoned that if cfDNA mediates immune regulatory activities, this could favor tumor immune escape, worsening the disease course, in tumor challenged-animals. Hence, the poly-C/poly-G oligonucleotide (10 µg/injection) was weekly administered for three weeks, starting from the day of tumor challenge to B16F10 melanoma-bearing C57BL mice. To verify whether even in this experimental model increased cfDNA concentrations were also associated with increased CD4 + Treg frequency, their frequency within the tumor infiltrate was analyzed in DNA-treated and untreated mice. Indeed, these experiments showed an increased frequency of intra-tumoral CD4 + Treg in poly-GC treated mice compared to control mice (Fig. 6I), an effect associated with accelerated tumor growth in DNA-treated than in untreated mice (*P* = 0.001) (Fig. 6 K). Interestingly, when the experiments were replicated in MHC class II KO C57BL mice, comparable tumor growth was observed in poly-GC treated and untreated mice (Fig. 6L). Collectively, these data further support the concept that cfDNA exert immune regulatory functions in vivo, fostering a pro-tumorigenic outcome in tumor challenged animals, a phenomenon that may be mediated, at least in part, by interactions between cfDNA and MHC class II molecules.

## Conclusion

Our study provides evidence that cfDNA-mediated immune regulatory activities in vitro and in vivo. cfDNA cellular and molecular targets have been identified in APC and MHC class II molecules, respectively, reminiscent of our pioneering observations on human cells [23, 24]. Our in-silico analysis identified a cfDNA high-affinity binding site within the grove of MHC class II molecules. Molecular, functional and in silico data, all together considered suggest that cfDNA may compete with antigenic peptides for binding to MHC class II molecules, explaining its capacity to inhibit antigen-specific T cell responses. Interestingly, we found a higher Kd of association of the Poly-C/PolyG to the I-Ab haplotype, with respect to that to I-Ad: this may occur because the I-Ab allele exhibits a broader binding capacity compared to that of I-Ad. This means it can bind a wider range of peptides, including those with less defined anchor residues. I-Ad is generally considered more restricted in its peptide binding repertoire compared to I-Ab [25, 26]. As Poly-C/Poly-G is a “non-peptide” ligand, it is more prone to bind to the less stringent. Moreover, we showed that macrophages exposed to cfDNA undergo gene expression changes favoring an anti-inflammatory behavior and Treg cell recruitment. In particular, we observed that PMJ2-PC macrophages treatment with poly-CG oligonucleotide induced reduced IL6 and increased IL10 productions as well as *EGR-1* and *CCL22* over expression. *Egr-1* regulates the LPS-induced inflammatory response by activating SOCS-1 gene expression and promotes macrophage polarization into anti-inflammatory (M2) subtype [27–29] which are able to inhibit the function of T and NK cells and to induce Treg cells [30–32]. *EGR1* repressive activity results in the suppression of inflammatory genes mediated by the NuRD corepressor complex [33, 34]. Hence, our findings, showing that cfDNA may upmodulate IL 10 secretion and *Egr-1* gene expression, suggest that cfDNA could foster anti-inflammatory effects and immune regulation of APC via intracellular signaling.

Concerning *CCL22*, increased gene expression levels were observed in poly-C/poly-G treated PMJ2-PC cells respect to untreated cultures, an effect concordant with that induced by LAG3-MHC class II interaction [35]. This finding is relevant since CCL22 is a chemokine strictly involved in Treg cells recruitment [36–40].

Hence, cfDNA-mediated immune regulatory effects may both be mediated by steric hindrance (through the possible displacement of antigenic peptides from the MHC class II grove) and intracellular signaling.

Evidence that cfDNA immune regulatory activity may have effects in vivo came from our studies on two ad hoc experimental models, one constituted by SLE-prone mice and the other one by an aggressive experimental melanoma. Concerning the SLE model, our data are in apparent conflict with the proposed mechanism which suggests that the formation of cfDNA-anti-DNA mAb immune complexes and cfDNA binding to TLR-9 are the main culprits in disease progression [39, 41]. Interestingly, our results show that increased cfDNA concentrations slow disease progression and that this effect is associated with increased frequency of splenic Treg cells, a phenomenon that may parallel the activating effects played by cfDNA on Breg cells [42]. This is a relevant finding since impairment of regulatory circuits has been observed in SLE and proposed as an initial step in the pathogenic cascade of SLE [43–45]. Concerning the tumor model, the finding that increased cfDNA concentrations favor tumor growth is not surprising since it provides a biological explanation for the observed inverse correlation between cfDNA concentrations and survival in cancer patients [46–50].

Overall, our findings may seem to contradict the common knowledge that considers cfDNA as a “danger signal” and the data that support the existence, among Pattern Recognition Receptors, of an array of DNA sensors, in the endosomal and cytosolic cell compartments, which can activate an innate immune response [50]. However, recent observations question the unique role of cfDNA as a danger signal mediated through TLR9 and STING. In fact, the TLR9 ablation in an MRL/lpr SLE mouse model led to accelerated disease onset [51]. Moreover, STING activation, through nanoparticle cargo DNA, protected mice from Ag-induced arthritis and experimental autoimmune encephalitis [52]– [53]. These observations and our original findings support a new concept: that cfDNA may have a dual function since it can activate regulatory pathways or can act as a danger signal. This double (pro- or anti-inflammatory) activity of cfDNA may depends on several factors as: (a) the concomitant presence of other danger signals (as in the case of silica-induced lung inflammation) [54]; (b) the frequency of CpG repeats within the molecule (as in bacterial DNA, favoring TLR9 activation); (c) the origin (i.e., mitochondrial vs. nuclear DNA, being the former highly enrich in CpG motifs like that of bacterial DNA) [55]; (d) the efficiency of clearance processes; (e) the site of interaction with endogenous molecular pattern (intra versus extracellular site). Therefore, additional studies are necessary to elucidate the precise conditions in which cfDNA effects lean towards regulatory instead of inflammatory functions, and the related mechanisms. Our current view is that cfDNA, physiologically originating from autologous cell death, must be tolerated. Thus, we anticipate that, in the absence of concomitant danger signals, cfDNA-mediated immune regulatory mechanisms (here described) prevail. On the contrary, pathogen-derived DNA, that usually is directly conveyed within the intracellular compartments by the infectious agent and it is associated with other danger signals, engages intracellular DNA sensors and ultimately mediates inflammation [56].

In sum, our study delineates a novel immune regulatory function for cfDNA, a feature independent from its genetic properties but linked to its interaction with specific immune receptors, i.e., MHC class II molecules.

## Experimental section

### Mice

Twelve weeks old NZBxNZWF1(BWF1) (I-A^d/u^/I-E ^d/u^), 8 weeks old BALB/c (I-A^d^/I-E^d^) and 8 weeks old C57BL/6J (I-A^b^/I-E^null^) mice were purchased from Envigo RMS (S.Pietro al Natisone, Udine, Italy) and housed under the specific pathogen–free conditions in the animal facility at the IRCCS A.O.U. San Martino – IST, Genoa, Italy.

Fifteen weeks old MHC class II KO C57BL/6J (B6.129S2-H2dlAb1-Ea/J) mice were purchased from The Jackson Laboratory (Bar Harbor, Maine, USA) and housed under the specific pathogen-free conditions at the Research Animal Resource Center (RARC) of Weill Cornell Medicine, New York, USA.

All the procedures were carried out by animal facilities qualified staff in accordance with the guidelines provided in Ministero della Salute D.lgs 26/2014. All experiments were performed on female mice.

### Cancer patients

Ten patients affected by metastatic (stage IV) prostate cancer were enrolled at the San Martino Polyclinic Hospital (Genoa, Italy). Heparinized blood samples (10 ml) were collected from each patient. The study was approved by the local ethics committee (NCT02293707); all patients and controls enrolled in the study provided written informed consent.

### Source of cfDNA and cfDNA dosage

As synthetic source of cfDNA, the following synthetic 20-mer oligonucleotides were synthetized by TIB Molbiol (Genoa, Italy) and then i.v. administered to experimental animals: oligo dC (5’- CCC CCC CCC CCC CCC CCC CC-3’), oligo dG (5’- GGG GGG GGG GGG GGG GGG GG-3’), oligo dA (5’- AAA AAA AAA AAA AAA AAA AA-3’), oligo dT (5’- TTT TTT TTT TTT TTT TTT TT-3’) and oligo dC Cy5.5 (5’ Cy5.5- GGG GGG GGG GGG GGG GGG GG-3’). Oligos (100 µM) were annealed to generate double strand (ds) poly-C/poly-G, poly-A/poly-T and Cy5.5 poly-C/poly-G or Alexa fluor 488 (AF488)-poly-C/poly-G in annealing buffer (50 mM Tris HCl, 10 mM MgCl_2_, 100 mM NaCl, 1mM DTT) by placing tube containing mixture in boiling water and allowing it to cool to room temperature.

As natural source of cfDNA, cfDNA from plasma of cancer patients was collected.

cfDNA was purified from plasma samples of both cancer patients and mice. In particular, mice were bled via retro-orbital plexus before and after 10 min from intravenous administration of the poly-C/poly-G oligonucleotide (10 µg/50 µl PBS/mouse). cfDNA purification was performed using NucleoSpin Plasma XS (Macherey-Nagel, Düren, Germany).

After its purification from plasma, cfDNA quantification was performed by Quant-it Picogreen dsDNA (Thermo Fisher Scientific, Waltham, MA) on an Infinite F200 Pro microplate reader (Tecan, Männedorf, Switzerland).

### Analysis of cfDNA/MHC class II molecules interaction by western blot

Splenocytes from BWF1 mice, expressing I-A^d/u^ I-E^d/u^ MHC II alloantigen, were lysed by 1% NP-40 cell lysis buffer with protease inhibitor cocktail (G-Biosciences, St Louis, MO). The lysates were run on a non-reducing SDS-polyacrylamide gel (Bolt 4–12% Bis-Tris Plus polyacrylamide gel, Thermo-Fisher Scientific). Then the gel was blotted on a nitrocellulose membrane and incubated either with a mouse anti mouse -I-A^d^ MHC class II mAb (160 ng/ml) or with a poly-C/poly-G oligonucleotide (30 ng/ml) followed by a mouse anti-dsDNA mAb (500 ng/ml) (Abcam, Cambridge, UK). The membrane was revealed with an anti-mouse IgG (Fc-specific)-peroxidase antibody (Sigma-Aldrich) (1:10000).

### Analysis of cfDNA/MHC class II molecules interaction by flow cytometry

Splenocytes derived from C57BL/6J mice were collected, minced and passed through a cell strainer (40 μm) to obtain a homogenous cell suspension. After red blood cell lysis (Red blood cell lysing buffer, Sigma Aldrich, St. Louis, MO) splenocytes were incubated or not with murine IL-2 (100 U) for 2 and 5 days. Mouse IL2 was added again after three days of incubation. Cells were pre-incubated 30 min in ice with anti CD16/32 antibody (Biolegend, San Diego, CA) to block the binding sites of these Fc-receptors, then incubated with the following fluorochrome-labeled monoclonal antibodies (mAbs): anti-CD3 labeled with Brilliant Violet (BV)510, anti-CD19 labeled with Peridinin chlorophyll protein-Cyanine5.5 (PerCP Cy5.5), anti-49b labeled with Fluorescein (FITC), anti-CD11b labeled with Phycoerythrin (PE)-Cy7, anti-MHC class II labeled with PE according to the manufactory instruction, in presence or not of Cy5.5 labeled poly-GC (100 ng). All mAbs were purchased from BD Biosciences, except for anti-49b FITC and anti-CD11b PE Cy7 that were purchased from eBiosciences.

Cells were acquired by a Flow cytometer BD FACSCanto II flow cytometer (Beckton Dickinson, BD Biosciences) using FACS DIVA software and analyzed by FlowJo (BD Biosciences) software.

Splenocytes from BWF1 mice administered or not with Cy5.5 conjugated poly-C/poly-G oligonucleotides and splenocytes from C57BL/6J mice were collected and analyzed by flow cytometry to identify the cellular subtype that preferentially binds cfDNA. To this aim, splenocytes were incubated (15 min at room temperature in dark condition) with the following fluorochrome-labeled mAbs: anti-CD3 BV510, anti-CD4 FITC, anti-CD25 PE Cy7, anti-CD19 PerCP Cy5.5, anti-49b FITC, anti-CD39 PerCP Cy 5.5, anti-CD11b PE Cy7, anti-CD45R FITC, anti–FoxP3 PE and anti-MHC class II PE according to the manufactory instruction. All mAbs were purchased from eBiosciences, except for anti-CD3 BV510, anti-CD19 PerCP Cy5.5 and anti-MHC class II PE that were purchased from BD Biosciences. After washings, the cells were analyzed by a Flow cytometer BD FACSCanto II flow cytometer (Beckton Dickinson, BD Biosciences) using FACS DIVA (BD Biosciences) software. Antibodies specifications are listed in Table S5.

### Bio-layer interferometry

The interaction between MHC class II molecules and the poly-C/poly-G oligonucleotide was characterized by Bio-layer Interferometry using a Fortébio Octet RED96e instrument (Sartorius).

Streptavidin coated BLI sensors (SA sensors) were purchased from FortéBio (Sartorius). Prior to ligand loading, biosensors were hydrated for at least 10 min in the loading buffer at room temperature, to dissolve the sucrose layer and for surface chemistry conditioning.

After an initial 30 s baseline check step the sensors were dipped for 10 min in biotinylated MHC-containing solutions prepared at 20 µg/mL and rinsed in buffer solution (Kinetic Buffer 1x, Fortébio, Sartorius) for 120 s. In these experiments, MHC class II I-A^d^ and I-A^b^ biotinylated monomers and an MHC class I H-2K^b^ biotinylated monomer, as control, (all kindly provided by NIH Tetramer Core Facility) were used. After the baseline step, the functionalized sensors were next dipped in different poly-C/poly-G oligonucleotide solutions prepared in Kinetic Buffer 1x (Fortébio, Sartorius) at different concentrations in the range 80 µM – 330 nM for 300 s followed by a 300 s dissociation step in the baseline buffer. Also, a reference sample of zero concentration was tested in order to correct for ligand loading drift. Reference sensors were prepared following the same experimental design but without ligand immobilization in order to correct for non‐specific binding on the biosensors surface chemistry layer. The sensorgrams were fitted by a heterogeneous model.

### Molecular modelling

Up to now, various crystallographic data of murine MHC class II have been obtained and released on Protein Data Bank, being only partially in agreement with those considered in these experiments. In particular, for molecular modelling calculations we selected the available crystallized proteins named by the pdb codes 1IEB (resolution = 2.7 Å) [57] and 1MUJ (resolution = 2.15 Å) [58], which correspond to the alfa and beta chains of poly-A/poly-T herein investigated.

The final MHC-II model employed in these studies was derived by matching the two aforementioned proteins with the aim at building a reliable three-dimensional structure of poly-A/poly-T, by means of the software MOE (MOE: Chemical Computing Group Inc. Montreal. H3A 2R7 Canada. http://www.chemcomp.com).

The coordinates of the complex were minimized and refined with the AMBER99 forcefield. Successively, aggregation-prone regions in the three models were calculated using the Protein Patch Analyser module implemented in MOE software. This application is used to generate visual representations of protein surface patches as a means for predicting locations highly prone to protein aggregation or for rationalizing protein-protein binding sites, being often related to the presence of hydrophobic interaction areas.

With this specific aim, recently a computational method called Spatial Aggregation Propensity (SAP) has been proposed, that is able to predict aggregation propensities by identifying hydrophobic surface patches [59].

The Protein Patch Analyzer tool implemented in MOE allows to determine the hydrophilic and hydrophobic patch regions of a specified protein. Three classes of surface patches are calculated on the basis of electrostatic and hydrophobic potential calculations, allowing for classification to hydrophobic, negative-charged and positive-charged patches. The hydrophobic potential is calculated applying the Wildman and Crippen LogP parameters [60].

Any calculated patch is therefore clearly classified in terms of several properties, such as the patch surface area (Å2), the patch surface as a percentage of the total surface area of the protein, the maximum potential value measured within the patch (kcal/mol) and the average patch potential (kcal/mol). In addition, the number of highly surface-exposed oxygen, nitrogen and sulfur atoms are determined as well, being probably related to the presence of residues with propensities for deamidation, or of surface-exposed tyrosine residues, which are of particular interest due to their ability to form covalent species.

In tandem with these analyses, the possibility of interaction between poly-A/poly-T and our murine model of MHC class II was preliminary scouted by running the site finder module of MOE, aimed at identifying the most probable protein cavities for binding contacts.

Finally, the putative binding mode underlining the key contacts between the aforementioned proteins was investigated by protein-protein docking calculations, by MOE software.

In protein-protein docking, one protein structure, referred to as the ligand, is docked against another, referred to as the receptor. Commonly, the smaller structure is chosen to be the ligand, but there is no requirement for it to be so. In antibody modeling, the antigen is usually specified to be the ligand, but again, there is no requirement for it to be so.

The protein-protein docker uses a multi-stage method for generating poses and then ranking them. Starting from a coarse-grained (CG) model to reduce the computational search space, a Fast Fourier Transform (FFT) approach is used to explore configuration space efficiently. This is followed by a minimization process that is built around a staged convergence protocol.

### OVA-specific T cell proliferation assay

Ovalbumin chicken egg grade V (OVA) (Sigma Aldrich, St. Louis, MO) and Complete Freund Adjuvant (CFA) (Sigma Aldrich, St. Louis, MO) were emulsified at 1:1 (vol/vol) ratio.

In 3 independent experiments, BALB/c mice were subcutaneously injected with the CFA emulsion containing OVA (60 µg) on days 0, 15 and 30.

Spleens of these OVA hyper-immune BALB/c mice were collected 2 weeks after the last OVA administration, minced and passed through a cell strainer (40 μm) to obtain a homogenous cell suspension. After red blood cell lysis (Red blood cell lysing buffer, Sigma Aldrich, St. Louis, MO) splenocytes were stained with carboxyfluorescein diacetate succinimidyl ester (CFDA-SE, Molecular Probes, Life Technologies, Segrate, MI, Italy) and seeded in triplicate wells at 10^5^ cells/well in a volume of 200 µl of RPMI medium (Gibco-Life Technologies) in the presence of autologous irradiated splenocytes (10^5^ cells/well) stained with Cell Trace (Thermo Fisher Scientific) and (or not, in negative control) OVA (1 ng/ml to 100 µg/ml). The experiments were also performed adding in the co-culture of CFDA-SE-labelled splenocytes and autologous irradiated splenocytes plus OVA either the poly-C/poly-G oligonucleotide (100 ng/ml) or cfDNA from cancer patients (2.5 ng/ml). To have a sufficient amount of cancer patient’s cfDNA for performing this analysis, aliquots of cfDNA from cancer patients #1 to #4, #5 to # 7 and #8 to #10 were separately pooled, achieving three different preparations of cfDNA. Cultures with medium alone or medium containing Concanavalin A (Con A, Sigma Aldrich) (final concentration 2.5 µg/ml) were used as negative and positive controls, respectively. Dead cells have been excluded by staining with 7-AAD (7-aminoactinomycin D, BD Biosciences). Proliferation of OVA-specific splenocytes was determined by CFDA-SE dilution using BD FACSCanto II flow cytometer (Beckton Dickinson, BD Biosciences) and FACS DIVA (BD Biosciences) software.

In other sets of experiments splenic APC enriched by T cells depletion performed using Dynabeads™ FlowComp™ Mouse Pan T (CD90.2) Kit, were stained with Cell Trace and pre-incubated with OVA peptide (100 ug/ml) for 2 h at 37 °C and 5% CO_2_. An antigen specific T cells proliferation test was settled down by incubating 2 × 10^5^ CFDA-SE-stained T cells purified from an OVA immunized mice with 2 × 10^5^ OVA pre-incubated APC in presence or not of poly-C/poly-G oligonucleotide (100 ng/ml). Dead cells have been excluded by staining with 7-AAD (7-aminoactinomycin D, BD Biosciences). Proliferation of OVA-specific splenocytes was determined by CFDA-SE dilution using BD FACSCanto II flow cytometer (Beckton Dickinson, BD Biosciences) and FACS DIVA (BD Biosciences) software.

### Mixed lymphocyte reactions

Splenocytes from BWF1 mice were stained with CFDA-SE and used as Responder, splenocytes from C57BL mice were stained with Cell trace, irradiated and used as Stimulator. 1 × 10^5^ Responder were incubated with 1 × 10^5^ Stimulator in the presence or not of poly-C/poly-G or poly-C/poly-G p (100 ng/ml). Dead cells have been excluded by staining with 7-AAD (7-aminoactinomycin D, BD Biosciences). Proliferation of BWF1 splenocytes was determined by CFDA-SE dilution using BD FACSCanto II flow cytometer (Beckton Dickinson, BD Biosciences) and FACS DIVA (BD Biosciences) software.

### CD3/CD28-mediate T cell proliferation

96 well plate was coated with anti-CD3 antibody (8ug/ml) and anti-CD28 (8ug/ml) and incubated ON at 4 °C overnight. T cells were separated by mouse spleen using Dynabeads™ FlowComp™ Mouse Pan T (CD90.2) Kit, stained with CFDA-SE and incubated at 2 × 10^5^ cells/well in presence or not of 2 × 10^5^ irradiated Cell Trace-stained APC, as stimulator and poly-C/poly-G oligonucleotide at different concentrations (100 ng/ml, 1 ng/ml, 0.0001 ng/ml). Dead cells have been excluded by staining with 7-AAD (7-aminoactinomycin D, BD Biosciences). Proliferation of T cells was determined by CFDA-SE dilution using BD FACSCanto II flow cytometer (Beckton Dickinson, BD Biosciences) and FACS DIVA (BD Biosciences) software.

### Enzyme-linked immunosorbent assay

RAW 264.7 cells (1 × 10^6^) were grown in DMEM 10% FCS in the presence or not of LPS (1 µg/ml) and in the presence or not of Poly-C/poly-G at different concentrations (100 ng/ml and 1 ng/ml) for 24 h at 37 °C and 5% CO_2_. Supernatants were collected and stored at −20 °C. IL-6 and IL-10 production were evaluated on supernatants by Quantikine ELISA Immunoassay (R&D Systems, Minneapolis, MN) following the manufacturer’s protocol.

### Binding inhibition test

5 × 10^5^ RAW 264.7 cells were grown in DMEM 10% FCS and pre-incubated for 10 min in ice with anti CD16/32 antibody (Biolegend, San Diego, CA) to block Fc-receptor binding. Then AF488-labeled poly-C/poly-G at different concentrations (10, 5, 1 or 0,5 µg/ml) in the presence or not of anti-mouse -I-A^d^ MHC class II mAb (2 µg/ml) (M5/114.15.2, BioLegend) or isotype control antibody was added and incubated for 30 min in ice. Cells were then washed with PBS and stained with LIVE/DEAD™ Fixable Aqua Dead Cell Stain Kit (Thermo Scientific) for 10 min at room temperature and washed in PBS and analyzed by a Flow cytometer BD FACSCanto II flow cytometer (BD) using FACS DIVA (BD) software.

Pmj2-PC (MHC-II high) and Pm2j-R (MHC-II low) macrophages cells were grown in DMEM 10% FCS and incubated with AF488-labeled Poly-C/Poly-G (100 ng/ml) for 1 h at 37 °C or with anti-mouse -I-A^d^ MHC class II mAb (2 µg/ml) (M5/114.15.2, BioLegend) (1 µg/ml) for 20 min at RT, cells were then washed with PBS and stained with LIVE/DEAD™ Fixable Aqua Dead Cell Stain Kit (Thermo Scientific) for 10 min at room temperature and washed in PBS and analyzed by a Flow cytometer BD FACSCanto II flow cytometer (BD) using FACS DIVA (BD) software.

### Analysis of gene expression

Pmj2-PC cells (10^6^) were incubated or not (controls) with different concentrations of poly-C/poly-G oligonucleotide. After 3, 6 and 24 h, cells were collected and RNA extraction was performed using Rneasy Plus Mini Kit (Qiagen, Hilden, Germany) according to the manufacturer’s instruction. RNA quality was evaluated at 260/280 ratio using Nanodrop (Thermo Fisher Scientific) and RNA integrity was assessed using Agilent Bioanalyzer 2100 (Agilent Technologies Inc., Santa Rosa, CA). RNA integrity number (RIN) values ranged from 7.5 to 10.0. One µg of RNA for each condition was retro-transcribed using QuantiTect Reverse Transcription kit (Qiagen) according to the manufacturer’s protocol. The described extraction procedure guaranteed complete removal of genomic DNA. A pre-designed panel of pathway-specific genes (Supplementary Table 3) was used to test the expression of 40 genes with Bio-Rad iQ5 real-time PCR system (Biorad, Hercules, CA) and analyzed by PrimePCR analysis Software Gene Study 1.0.030.1023.

All experiments were performed with SYBR Green Supermix and 96-well plates (Biorad, Hercules, CA) at 60 °C annealing temperature. *GAPDH* was used as an endogenous reference to normalize gene expression values with the 2^−ΔΔCt^ method [61]. A fold change of ≥ 2.0 or ≤ 0.5 and a p-adjusted value of < 0.05 were considered to be significantly up or down regulated. To confirm the significant modulation of the differentially expressed genes resulting from Bio-Rad iQ5 real-time PCR analysis, we performed real-time quantitative PCR using Light cycler nano (Roche Diagnostics, Mannheim, Germany) and specific primer (Supplementary Table 6). We also analyzed expression level of CCL-22 gene not included in the Bio-Rad iQ5 pre-designed panel, by Light cycler nano. *TATA-Box Binding Protein* housekeeping gene was used as internal control and all experiments were repeated three times.

### Bioinformatic analysis

In silico protein–protein functional interactions among the differentially expressed genes were assessed with the STRING database (http://stringdb.org, accessed on 27 April 2023) [18] using default parameters. An extension of the network was analyzed to assess potential indirect interactions between the differentially expressed genes.

### DNA administration to BWF1 SLE-prone mice

Twenty weeks old BWF1 mice were administered with DNA when their mean proteinuria level scored 1 (corresponding to 30 mg/dl). Mice were divided in three groups (10 mice per group) to be administered either with poly-C/poly-G oligonucleotide (10 µg in 50 µl PBS), poly-A/poly-T oligonucleotide (10 µg in 50 µl PBS) or only PBS (control mice). Proteinuria was assessed in all groups of mice before treatment and at weekly intervals after the beginning of the treatment using Albustix strips (Siemens, Munich, Germany). Mice were sacrificed when proteinuria reached level > 3 (corresponding to > 300 mg/dl) or mice developed ascites. The experiment was repeated 2 times.

In order to perform ex vivo imaging, BWF1 mice were i.v. injected with either Cy5.5 poly-C/poly-G (4 mice) or poly-A/poly-T oligonucleotides (2 mice) and sacrificed 1 h after DNA administration. Organs from poly-C/poly-G and control mice were collected and analyzed by In Vivo Imaging System (IVIS) scanning (Perkin-Elmer, Walthman, MA).

### DNA administration to melanoma-challenged C57BL/6J mice

Ten weeks old C57BL/6J mice were subcutaneously challenged with B16F10 cells (10^5^/100 µl PBS). Seven of these melanomas challenged mice were i.v. injected with the poly-C/poly-G oligonucleotide (10 µg/100 µl PBS) once a week for three weeks starting from the day of tumor challenge. Control mice (7 mice) received only PBS. Tumor size was daily monitored using a caliper and tumor volumes were calculated according to the formula: (a2*b)/2, where a is the shorter and b is the longer tumor diameter. Mice were sacrificed either when tumor reached a volume of 1 cm^3^ or in the presence of tumor ulceration. The experiment was repeated 2 times.

Fifteen weeks old MHC class II KO C57BL mice were subcutaneously challenged with B16F10 cells (10^5^/100 µl PBS). Five of these melanomas challenged mice were i.v. injected with the poly-C/poly-G oligonucleotide (10 µg/100 µl PBS) once a week for three weeks starting from the day of tumor challenge. Control mice (5 mice) received only PBS. Tumor size was daily monitored using a caliper and tumor volumes were calculated as above. Mice were sacrificed when tumor reached a volume of 1 cm^3^. The experiment was performed once.

### Statistical analyses

Statistical analysis was performed using Prism 5.04 software from GraphPad Software (La Jolla, CA). For all distributions, the D’Agostino and Pearson omnibus test was used to assess normality. Parametric or nonparametric tests were applied consequently. For multiple comparison, *P* values were calculated by one way or 2way Anova test. For survival analysis *p* values were calculated using Log-Rank Mantel Cox test. Only for comparisons reaching significance (α = 0.05) the *P* value was shown.

## Supplementary Information

Below is the link to the electronic supplementary material.

Supplementary Material 1

## Data Availability

The datasets generated during the current study are available from the corresponding author on reasonable request.
