# Supplementary material for: Immunoregulatory properties of cell free DNA
Source: Cell Mol Life Sci. 2025 Aug 26;82(1):320. doi: 10.1007/s00018-025-05862-y (PMC12381308; doi:10.1007/s00018-025-05862-y)
Supplement: Supplementary file 1 — Supplementary Material 1 [file 18_2025_5862_MOESM1_ESM.pdf]

Supporting Informations

Supplementary Figure 1

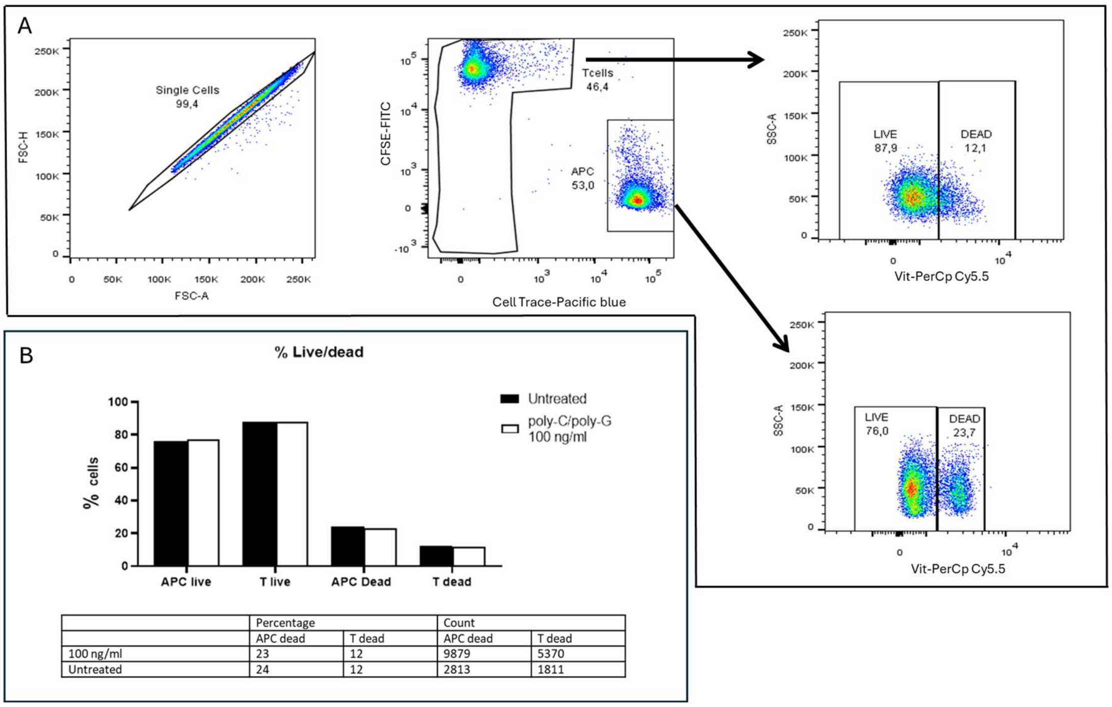

	Percentage		Count	
	APC dead	T dead	APC dead	T dead
100 ng/ml	23	12	9879	5370
Untreated	24	12	2813	1811

Fig. S1 Gating strategy and live/dead estimation
(A) Gating strategy on a representative control sample
(B) Live/dead estimation on untreated (black bar) or Poly-C/poly-G (100 ng/ml) treated T cells and APCs (white bar)

Supplementary Figure 2

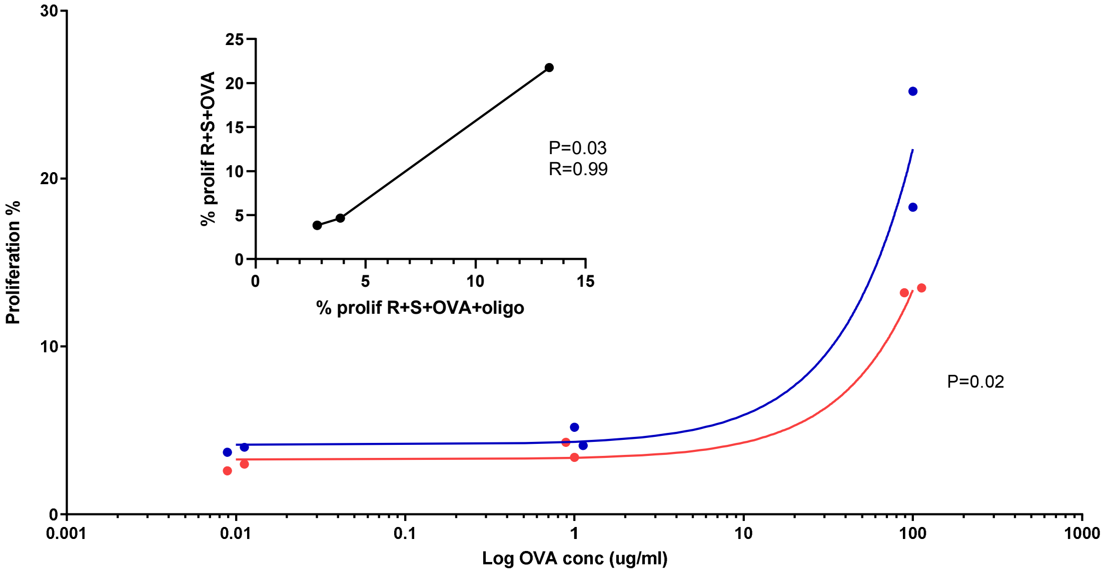

Fig. S2 Dose-response antigen specific proliferation and Poly-C/poly-G inhibition
The main graph shows the results of OVA-specific proliferation assays performed with splenocytes from different OVA-immunized BALB/c mice incubated with irradiated autologous splenocytes plus OVA at different concentrations (1ng/ml to 100 µg/ml) (blue line); irradiated autologous splenocytes plus OVA at different concentrations (1ng/ml to 100 µg/ml) and the poly-C/poly-G oligonucleotide (100 ng/ml) (red line); The inserted graph shows correlation between the two slopes.

Supplementary Figure 3

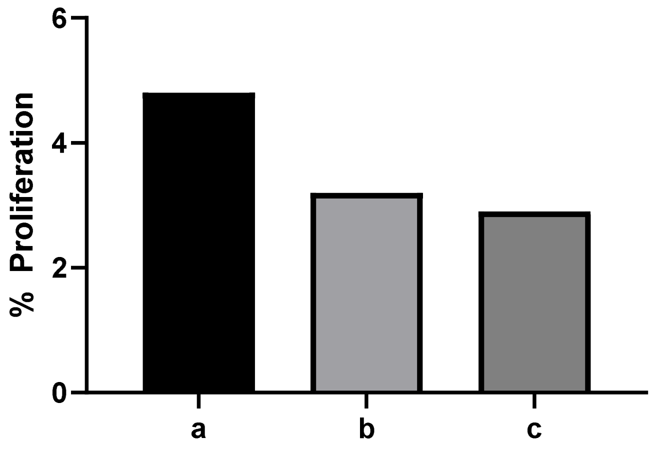

FIG S3: Mixed lymphocyte reaction
Representative MLR assay performed with splenic cells isolated from a BWF1 mouse stimulated with: (a) irradiated splenocytes from a C57BL mouse; (b) irradiated splenocytes from a C57BL mouse and the poly-A/poly-T oligonucleotide (100 ng/ml); (c) irradiated autologous splenocytes plus OVA and the poly-C/poly-G oligonucleotide (100 ng/ml);

Supplementary Figure 4

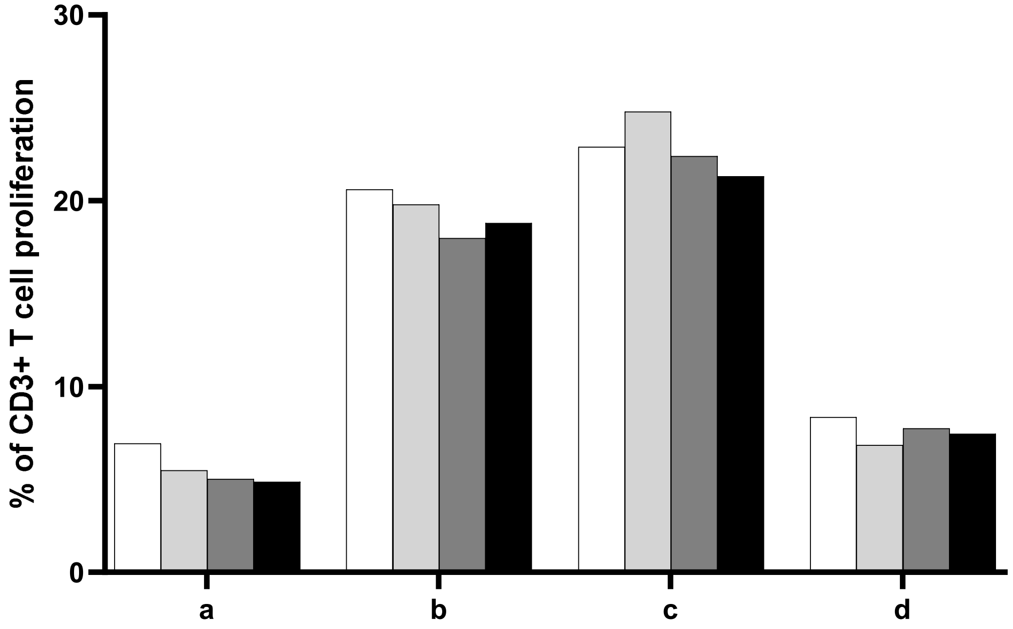

Fig. S4 CD3/CD28-mediate T cell proliferation and Poly-C/poly-G inhibition
The graph shows the results of CD3/CD28-mediate T cell proliferation assay performed with splenic T cells from OVA-immunized BALB/c (a), incubated with anti-CD3/CD28 mAb (b), or with irradiated autologous splenocytes plus anti-CD3/CD28 mAb (c), or with irradiated autologous splenocytes alone (d) untreated (white bar) or treated with poly-C/poly-G oligonucleotide at different concentrations: 0.0001 ng/ml (light grey bar), 1 ng/ml (dark grey bar) and 100 ng/ml (black bar).

Supplementary Figure 5

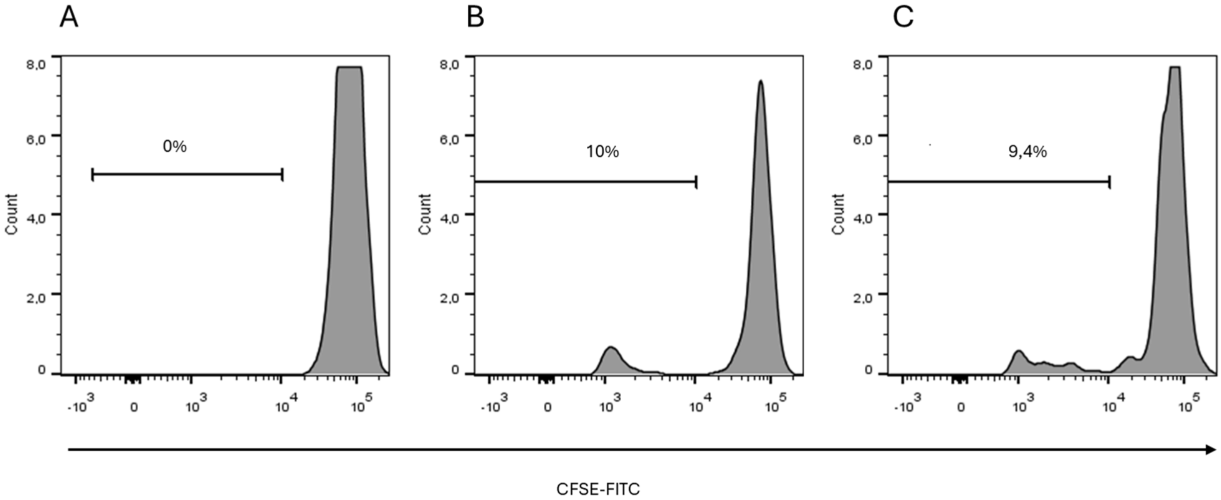

FIG S5: Antigen blocks Poly-C/poly-G binding to MHC class II
Representative histograms showing T cell proliferation using APC preincubated with OVA peptide: (A) T cells plus OVA; (B) T cells plus APC preincubated with OVA; (C) T cells plus APC preincubated with OVA and poly-C/poly-G oligonucleotide.

Supplementary Figure 6

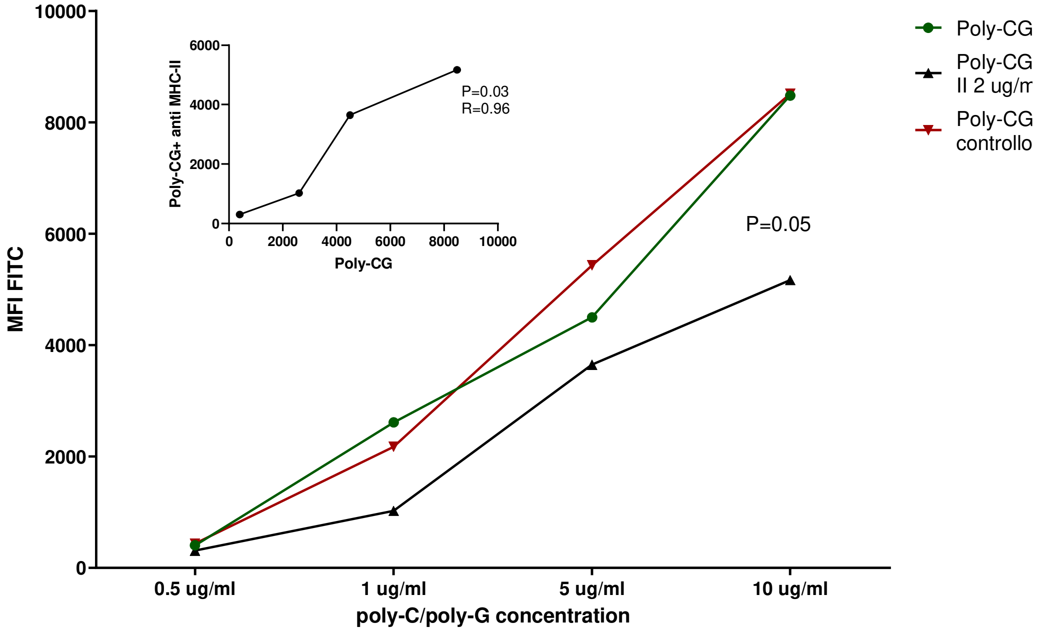

FIG S6: Poly-C/poly-G competes with MHC class II mAb for MHC class II binding
The main graph shows the mean fluorescence intensity (MFI) of RAW cells treated with (AF488)-labeled poly-C/poly-G (green line), AF488-labeled poly-C/poly-G plus anti-mouse -I-A^d MHC class II (black line) or (AF488)-labeled poly-C/poly-G plus isotype control antibody (red line). The inserted graph shows the correlation between the green and black slopes.

Supplementary Figure 7

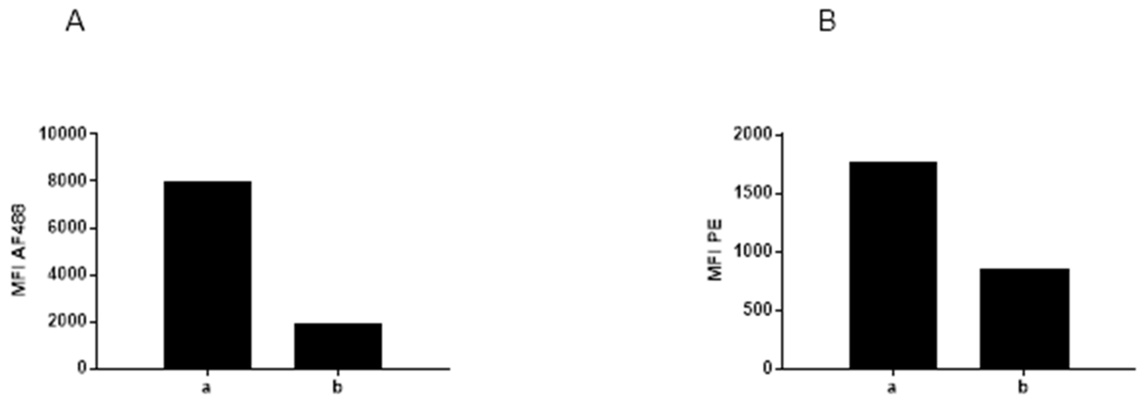

FIG S7: Poly-C/Poly-G binds preferentially to class II+ PMJ-PC cells respect to class II- PMJ-R cells.
MFI of PMJ-PC (a) and PMJ-R (b) macrophages incubated with AF488-labeled Poly-C/Poly-G (A) or antibody anti-MHC-II PE (B)

Supplementary Table 1. Effects of cfDNA from cancer patients on OVA-specific T cell proliferation.

cfDNA preparation n.	OVA* % of proliferating T cells	OVA + cfDNA** % of proliferating T cells (% inhibition)
1 (from cancer patient #1 to #4)	25%	1% (96%)
2 (from cancer patient #5 to #7)	16%	0.1% (99%)
3 (from cancer patient #8 to #10)	18%	2.5% (86%)

* Co-cultures of splenocytes from OVA hyper-immune BALB/c mice with autologous irradiated splenocytes plus OVA; ** Co-cultures of splenocytes from OVA hyper-immune BALB/c mice with autologous irradiated splenocytes plus OVA in the presence of different pools of cfDNA from cancer patients. Data are expressed as percentages of proliferating T cells

Supplementary Table 2. BLI results applying heterogeneous model binding 2:1 to cfDNA-MHC interaction.

Parameter	MHC monomer		
	I-A(d)	I-A(b)	H-2K(b)
**KD* (M)**	5,70E-06	8,54E-06	7,02E-05
**KD2**	7,52E-05	4,79E-05	5,82E-06
**KD ERROR**	2,46E-07	2,48E-07	1,31E-05
**KD2 ERROR**	1,15E-06	1,11E-06	1,97E-06
**KA** (1/MS)**	1,79E+02	1,68E+02	3,82E+02
**KA2**	5,15E+00	8,07E+02	6,59E+01
**KA2 ERROR**	6,69E+00	1,73E+01	6,96E+03
**KDIS*** (1/S)**	1,02E-03	1,43E-03	2,68E-02
**KDIS2**	3,45E-02	3,87E-02	1,25E-01
**KDIS ERROR**	3,30E-05	3,16E-05	1,90E-03
**KDIS2 ERROR**	1,66E-04	3,41E-04	1,23E-02
**FULL X2********	0,1088	0,1215	0,9046
**FULL R^2***********	0,9923	0,9867	0,7299
**KD (%)**********	9	29	78
**KD2 (%)**	91	71	22

*KD: Dissociation constant; ** KA: Association constant; ***KDIS: dissociation rate constant; ****FULL X2: the chi-square (measuring how a model compares to actual observed data); ***** FULL R^2: the statistical measure of how close the data are to the fitted regression line; ******KD(%): the percentage for which that interaction domain competes for the global affinity of the interaction.

Supplementary Table 3. Differentially expressed genes

Nr	Gene symbol	Gene name	Seq. name	2Log Fold Change	p.value	Regulation
1	Calm1	Calmodulin 1	NM_009790.5	1,48	0,017488	up
2	Calm2	Calmodulin 2	NM_001355703	1,77	0,000079	up
3	Calm3	Calmodulin 3	NM_007590	1,54	0,036058	up
4	Egr1	Early growth response protein 1	NM_007913.5	1,93	0,000059	up
5	Egr2	Early growth response protein 2	NM_001347458.1	1,48	0,09974	up
6	Egr3	Early growth response protein 3	NM_001289925.2	1,36	0,001103	up
7	Grb2	growth factor receptor bound protein 2	NM_001313936.1	2,19	0,000741	up
8	Gsk3a	glycogen synthase kinase 3 alpha	NM_001031667.1	1,87	0,078208	up
9	Gsk3b	glycogen synthase kinase 3 beta	NM_001347232.1	1,66	0,083353	up
10	Hras1	Harvey rat sarcoma virus oncogene 1	NM_001130443.2	-1,37	0,049845	down
11	Ikbkb	inhibitor of kappaB kinase beta	NM_001159774.1	2,35	0,029791	up
12	Ikbkg	inhibitor of kappaB kinase gamma	NM_001136067.2	1,9	0,022403	up
13	Jun	jun proto-oncogene	NM_010591.2	1,21	0,028457	up
14	Lyn	LYN proto-oncogene	NM_001111096.2	1,7	0,012767	up
15	Mapk1	mitogen-activated protein kinase 1	NM_001038663.1	1,34	0,056682	up
16	Mapk14	mitogen-activated protein kinase 14	NM_001168508.1	-1,72	0,044034	down
17	Mapk3	mitogen-activated protein kinase 3	NM_011952.2	1,62	0,098875	up
18	Mapk8	mitogen-activated protein kinase 8	NM_001310452.1	1,24	0,000132	up
19	Nfatc1	nuclear factor of activated T cells 1	NM_198429.2	1,62	0,008236	up
20	Nfatc2	nuclear factor of activated T cells 2	NM_001291172.1	1,35	0,017991	up
21	Nfatc3	nuclear factor of activated T cells 3	NM_001368796.1	1,37	0,009712	up
22	Nfkb1	nuclear factor of kappa light polypeptide gene enhancer in B cells 1	NM_001410442.1	1,39	0,090489	up
23	Nfkb2	nuclear factor of kappa light polypeptide gene enhancer in B cells 2	NM_001177369.1	1,37	0,254105	up
24	Nfkbia	nuclear factor of kappa light polypeptide gene enhancer in B cells inhibitora	NM_010907.2	1,86	0,003446	up
25	Nfkbib	nuclear factor of kappa light polypeptide gene enhancer in B cells inhibitor b	NM_001306222.1	1,18	0,303611	up
26	Nfkbie	nuclear factor of kappa light polypeptide gene enhancer in B cells 1b	NM_001410442.1	2,13	0,015143	up
27	Orai1	ORAI calcium release-activated calcium modulator 1	NM_175423.3	-1,15	0,677056	up

28	Pik3ca	phosphoinositide-3-kinase catalytic subunit a	NM_006218.4	1,67	0,023905	up
29	Pik3cb	phosphoinositide-3-kinase catalytic subunit b	NM_029094.3	1,36	0,000572	up
30	Pik3cd	phosphoinositide-3-kinase catalytic subunit d	NM_008840.3	1,1	0,03582	up
31	Pik3r1	phosphoinositide-3-kinase regulatory subunit 1	NM_001024955.2	1,2	0,100768	up
32	Plcg1	phospholipase C, gamma 1	NM_021280.3	1,31	0,132333	up
33	Plcg2	phospholipase C, gamma 2	NM_172285.2	1,57	0,000131	up
34	Ppp3ca	protein phosphatase 3, catalytic subunit, alpha isoform	NM_001293622.1	1,55	0,029161	up
35	Ptgs2	prostaglandin-endoperoxide synthase 2	NM_011198.5	1,26	0,007176	up
36	Raf1	v-raf-leukemia viral oncogene 1	NM_001356333.2	1,74	0,001319	up
37	Sos1	SOS Ras/Rac guanine nucleotide exchange factor 1	NM_009231.2	1,03	0,712345	up
38	Sos2	SOS Ras/Rac guanine nucleotide exchange factor 2	NM_001135559.1	1,17	0,049447	up
39	Stim1	stromal interaction molecule 1	NM_001374058.1	1,39	0,015939	up
40	Syk	spleen tyrosine kinase	NM_001198977.1	1,41	0,014851	up

Supplementary Table 4. cfDNA plasma concentrations.

	Before poly-GC injection	After poly-GC injection
Mouse 1	16.14*	166.39
Mouse 2	16.66	275.97
Mouse 3	35.91	119.57

*cfDNA concentrations are expressed as ng/ml.

Supplementary Table 5. Antibodies.

Target	Clone	Conjugation	Provider
I-Ad MHC class II	39-10-8		BioLegend
CD3	17A2		eBioscience
CD28	3751		BD Pharmigen
dsDNA	3519		Abcam
IgG (Fc-specific)	---	HRP	Sigma-Aldrich
CD16/32	2.4G2		BD Pharmigen
CD3	145-2C11	BV510	BD Horizon
CD4	GK 1.5	FITC	eBioscience
CD19	1D3	PerCP Cy5.5	BD Pharmigen
CD25	PC 61.5	PE Cy7	eBioscience
CD39	24DM51	PerCP Cy 5.5	eBioscience
CD45R	3-6B2	FITC	Tonbo Biosciences
CD49b	DX5	FITC	eBioscience
CD11b	M1/70	PE Cy7	eBioscience
IA-IE MHC class II	M5/114.15.2	PE	eBioscience
FoxP3	FJK-165	PE	eBioscience

Supplementary Table 6. Primers.

Gene	Forward primer sequence	Reverse primer sequence
EGR1 F	5'-TGTGATGACTCTGCTGTGAC-3'	5'-CACTCTGACACATGCTCCAGT-3'
IKBKB	5'-GGCACCTTGGATGACCTAGA-3'	5'-CCATATCCTGGCTGTCACCT-3'
MAPK14	5'-TGGCCCTGCCTTTACCATATC-3'	5'- ATGCATGGCTGAGGGATAGC-3'
NFKBie	5'-TGGCTGAGGACCTCGGATG-3'	5'-CAGATCGGCTCTTCCTCGTC-3'
GRB2	5'-CCGAGCGAACAACCCTATGA -3'	5'- AGGCTGAAAAGGGGTTCAGG -3'
CCL22	5'- CTG ATG CAG GTC CCT ATG GT-3'	5'- TTG CGG CAG GAT TTT GAG GT-3'
Tbp	5'- GTT GGG CTT CCC AGC TAA GT-3'	5'- GCT CAT AGC TAC TGA ACT GCT G-3'
GAPDH	5'- GGC ATC CTG GGC TAC ACT GA-3'	5'- TGG TGG TCC AGG GGT CTT-3'
